# Systematic review of urological injury during caesarean section and hysterectomy

**DOI:** 10.1007/s00192-022-05339-7

**Published:** 2022-10-17

**Authors:** Gavin Wei, Frances Harley, Michael O’Callaghan, James Adshead, Derek Hennessey, Ned Kinnear

**Affiliations:** 1 Department of Urology, Austin Health, Melbourne, Australia; 2 Department of Urology, Western Health, Melbourne, Australia; 3 Adelaide Medical School, University of Adelaide, Adelaide, Australia; 4 Urology Unit, Flinders Medical Centre, Bedford Park, Adelaide, Australia; 5 Flinders University, Adelaide, Australia; 6 Lister Hospital, Stevenage, UK; 7 Department of Urology, Mercy University Hospital, Cork, Ireland

**Keywords:** Bladder, Ureter, Caesarean, Hysterectomy, Iatrogenic, Injury

## Abstract

**Introduction and hypothesis:**

We aim to review iatrogenic bladder and ureteric injuries sustained during caesarean section and hysterectomy.

**Methods:**

A search of Cochrane, Embase, Medline and grey literature was performed using methods pre-published on PROSPERO. Eligible studies described iatrogenic bladder or ureter injury rates during caesarean section or hysterectomy. The 15 largest studies were included for each procedure sub-type and meta-analyses performed. The primary outcome was injury incidence. Secondary outcomes were risk factors and preventative measures.

**Results:**

Ninety-six eligible studies were identified, representing 1,741,894 women. Amongst women undergoing caesarean section, weighted pooled rates of bladder or ureteric injury per 100,000 procedures were 267 or 9 events respectively. Injury rates during hysterectomy varied by approach and pathological condition. Weighted pooled mean rates for bladder injury were 212–997 events per 100,000 procedures for all approaches (open, vaginal, laparoscopic, laparoscopically assisted vaginal and robot assisted) and all pathological conditions (benign, malignant, any), except for open peripartum hysterectomy (6,279 events) and laparoscopic hysterectomy for malignancy (1,553 events). Similarly, weighted pooled mean rates for ureteric injury were 9–577 events per 100,000 procedures for all hysterectomy approaches and pathologies, except for open peripartum hysterectomy (666 events) and laparoscopic hysterectomy for malignancy (814 events). Surgeon inexperience was the prime risk factor for injury, and improved anatomical knowledge the leading preventative strategy.

**Conclusions:**

Caesarean section and most types of hysterectomy carry low rates of urological injury. Obstetricians and gynaecologists should counsel the patient for her individual risk of injury, prospectively establish risk factors and implement preventative strategies.

**Supplementary information:**

The online version contains supplementary material available at 10.1007/s00192-022-05339-7.

## Introduction

Iatrogenic injury of the bladder or ureter is a known complication of abdominal, pelvic or vaginal surgery. Potential sequelae include haemorrhage, sepsis, renal loss and death [1–3]. The majority of such injuries occur secondary to caesarean section and hysterectomy [2, 4, 5], with a rising proportion now due to ureteroscopy [6, 7]. However, there remains great variation in the estimation of the frequency of urological injury during these major obstetric and gynaecological procedures, which limits the mandate for quality improvement exercises. Therefore, the objective of this review is to determine the incidence of urological injury during caesarean section and each type of hysterectomy.

## Materials and methods

### Search strategy

**Fig. 1:**
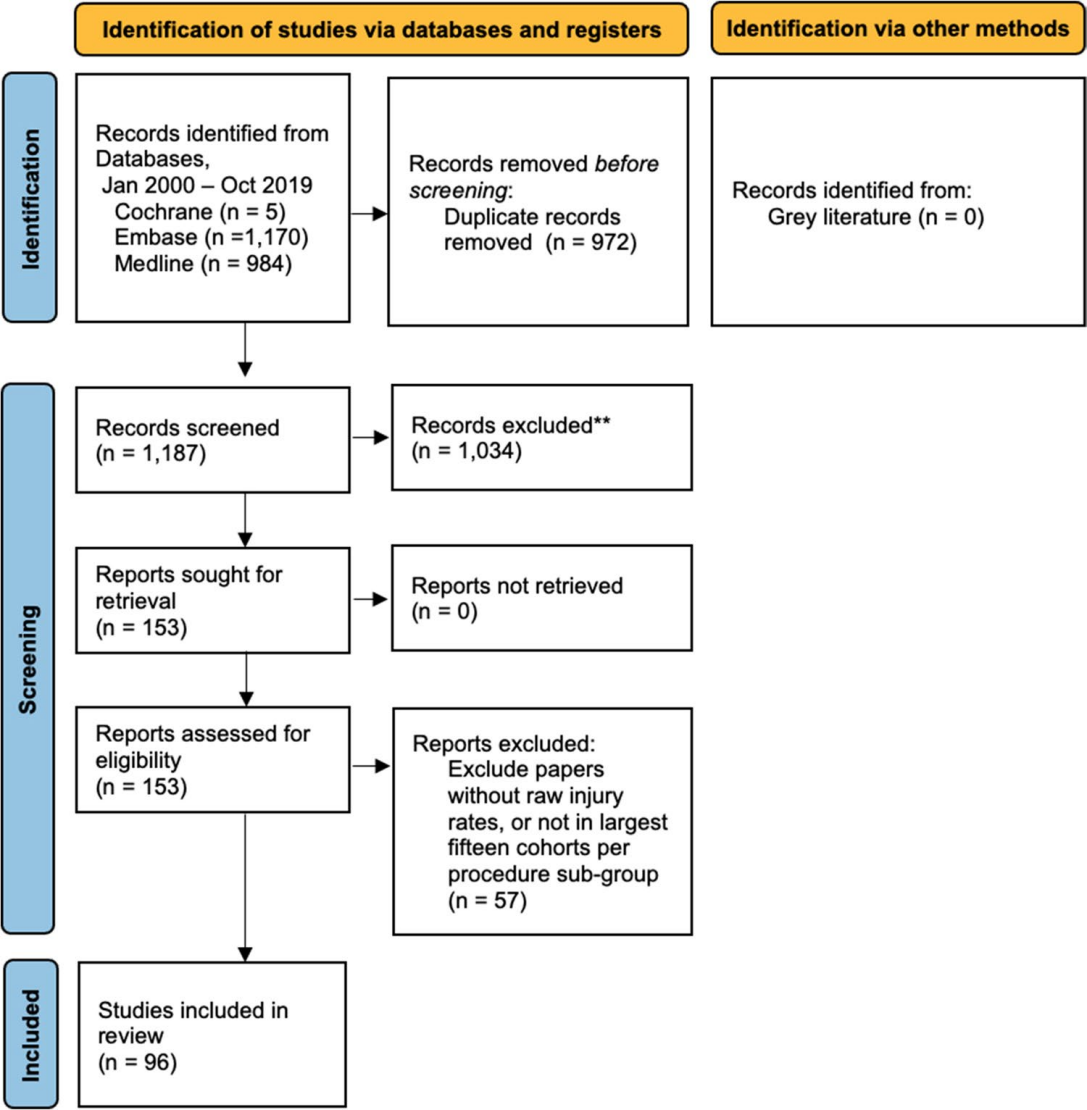
Preferred Reporting Items for Systematic reviews and Meta-Analyses flow diagram. **Based on title/abstract screen against study eligibility criteria

Systematic searches were performed of the Cochrane Central Register of Controlled Trials (CENTRAL), Embase and Medline. Searches were performed by Title or Abstract, utilising keywords and Boolean operators as follows: (obstetric, gynaecolog*, gynecolog*, caesarean OR hysterectomy) AND (urolog*, kidney, renal, ureter, bladder OR urethra) AND (iatrogenic, accidental, inadvertent, injur* OR trauma). Grey literature was also searched and eligible, by review of the above search results, bibliographies of retrieved articles and proceedings of the 2010–2019 annual scientific conventions of the Royal Australasian College of Surgeons. Inclusion criteria were agreed upon by all authors. Our method for identifying and evaluating data complied with the Preferred Reporting Items for Systematic Reviews and Meta-analyses criteria [8] (Appendix 1, Fig. 1). This included pre-publication of our intended analysis on PROSPERO (CRD42020161389). Note that although this protocol was intended to restrict inclusion to studies of ≥100 women, this was later reduced to ≥50 women, to reduce instances where identification of insufficient studies precluded meta-analysis. After protocol publication, preventative measures was demoted to a secondary outcome, to allow the study to focus on injury incidence as the sole primary outcome. Identified studies were screened by title and abstract, followed by full-text review. Articles then progressed to data extraction, including review of references. Two independent authors performed study screening and data extraction using a pre-defined form, with a third author involved for instances of disagreement (Appendix 2). Data extraction was performed twice to confirm accuracy. The final list of included articles was determined by compliance with the inclusion criteria and with the consensus of all authors.

### Study eligibility

Study eligibility was determined utilising the patient population, intervention, comparator, outcome and study (PICOS) method [9]. Eligible studies reported cohorts of ≥50 women (P) undergoing open or laparoscopic pelvic or abdominal surgery by an obstetrician/gynaecologist (I), were not required to have a comparator cohort (C) and stated raw incidence of iatrogenic injury to the urinary bladder or ureter (O). Eligible publications were original full-length articles, published in English between 01 January 2000 and 20 October 2019 (S). All databases and sources were last searched on 30 November 2019. Studies were collated and analysed separately based on procedure type (caesarean section, hysterectomy), access (open trans-abdominal, open vaginal, laparoscopic trans-abdominal, laparoscopically assisted vaginal, vaginal only, robot assisted) and histology (benign, malignant, any). Studies were ranked by cohort size. When >15 studies were identified within a given sub-group, only the largest 15 studies were included. This limitation, not prespecified in the Prospero protocol, was to ensure that meta-analyses were of manageable size.

### Intended analyses

The primary outcomes were the incidence of iatrogenic bladder and ureteric injury during caesarean section and each type of hysterectomy. Secondary outcomes were identified risk factors for injury and suggested preventative measures.

Qualitative summary was intended for all data, tabulating the key features of included studies. Raw proportions of each injury type were combined in a random effects meta-analysis using the R package “meta” [10]. Studies with zero events were not excluded from this pooled analysis. All analyses were two-tailed and significance was assessed at the 5% alpha level. Injury rates were reported as events per 100,000 procedures.

### Bias

The authors did not anticipate identifying any randomised controlled trials. Consequently, risk of bias was assessed with the Newcastle–Ottawa Scale, in accordance with the Cochrane Handbook [11, 12]. Each study was independently reviewed by two reviewers (GW, NK) against pre-defined criteria (Appendix 3). Instances of disagreement were resolved by consensus. Risk of bias was not used to exclude studies.

## Results

Initial database searches returned 2,159 articles. After removing 972 duplicate results and a further 1,034 irrelevant publications based on title and abstract review, 153 articles were retrieved for full text review (Appendix 4). After excluding ineligible studies and applying the pre-specified limitation of the 15 largest cohorts in each procedure sub-category, 96 eligible articles were included, describing caesarean section or hysterectomy in 130 cohorts, totalling 1,741,894 women (Fig. 1, Table 1) [3, 13–107].

**Table 1:** Enrolled studies

Year	Author	Nation	Procedure	Design	Sites	Cases	Bladder injuries	Ureteric injuries	Age	BMI	Exclusions	Follow-up (months)
2000	Conde-Agudelo [13]	Colombia	Abdominal hysterectomy (benign)	Prospective	Single	867	3	1	43	–	Cancer	45^a^
2001	Cosson et al. [14]	France	Abdominal hysterectomy (benign)	Retrospective	Multiple	166	3	0	–	–	Cancer	–
			Vaginal hysterectomy (benign)	Retrospective	Multiple	1,248	11	0	–	–	Cancer	–
2001	Leanza et al.[15]	Italy	Abdominal hysterectomy (mixed)	Retrospective	Single	2,765	4	4	–	–	–	–
			Vaginal hysterectomy (Mixed)	Retrospective	Single	373	2	1	–	–	–	–
2001	Liapis et al. [16]	Greece	Abdominal hysterectomy (mixed)	Retrospective	Single	3,741	–	13	–	–	–	–
			Vaginal hysterectomy (mixed)	Retrospective	Single	1,381	–	0	–	–	–	–
2001	Mathevet et al. [17]	France	Vaginal hysterectomy (benign)	Retrospective	Single	3,076	54	1	48	–	–	–
2001	Milad et al. [18]	USA	Laparoscopic assisted vaginal hysterectomy (benign)	Retrospective	Single	105	3	0	47	–	Simultaneous procedures	–
2001	Seman et al. [19]	Australia	Laparoscopic hysterectomy (mixed)	Retrospective	Single	436	–	6	–	–	–	–
2002	Wattiez et al. [20]	France	Laparoscopic hysterectomy (benign)	Retrospective	Single	1,647	19	6	–	–	Total uterine prolapse, cancer	–
2003	Boukerrou et al. [21]	France	Vaginal hysterectomy (benign)	Prospective	Multiple	741	10	0	46	–	–	–
2003	Khashoggi [22]	Saudi Arabia	Caesarean section	Retrospective	Single	290	3	0	34	31	<2 prior LSCS, missing data	–
2003	Shen et al. [23]	Taiwan	Laparoscopic assisted vaginal hysterectomy (mixed)	Retrospective	Single	2,702	11	4	46	–	–	–
2004	Dorairajan et al. [24]	India	Abdominal hysterectomy (benign)	Retrospective	Single	2,095	4	5	–	–	–	–
			Abdominal hysterectomy (malignant)	Retrospective	Single	617	3	1	–	–	–	–
			Vaginal hysterectomy (benign)	Retrospective	Single	3,270	10	0	–	–	–	–
2004	Koh et al. [25]	Taiwan	Laparoscopic assisted vaginal hysterectomy (mixed)	Retrospective	Single	2,006	5	0	45	–	–	1^b^
2004	Parkar et al. [26]	Egypt	Laparoscopic assisted vaginal hysterectomy (mixed)	Retrospective	Single	149	6	0	–	–	Missing data	–
2004	Rashid and Rashid [27]	Saudi Arabia	Caesarean section	Retrospective	Single	308	4	–	35	34	<5 prior LSCS, incomplete records	–
2004	Steed et al. [28]	Canada	Abdominal hysterectomy (malignant)	Prospective	Single	205	7	1	44	–	Lymph node metastases	21^a^
2005	Chang et al. [29]	Taiwan	Laparoscopic assisted vaginal hysterectomy (benign)	Retrospective	Single	225	2	0	46	23	–	12^c^
2005	Phipps et al. [30]	USA	Caesarean section	Retrospective	Single	14,757	42	–	31	32	–	–
2005	Tae Kim et al. [31]	South Korea	Abdominal hysterectomy (malignant)	Retrospective	Single	338	0	1	43	23	–	–
2005	Vakili et al. [32]	USA	Abdominal hysterectomy (benign)	Prospective	Multiple	278	7	6	42	31	Cancer	–
2006	Akyol et al. [33]	Turkey	Vaginal hysterectomy (benign)	Retrospective	Single	886	22	1	53	–	Nil	–
2006	Bojahr et al. [34]	Germany	Laparoscopic hysterectomy (benign)	Retrospective	Single	1,706	3	1	46	25	Cancer, endometriosis	–
2006	Dauleh et al. [35]	Qatar	Caesarean section	Retrospective	Single	21,337	16	4	–	–	–	–
2006	Ghezzi et al. [36]	Italy	Laparoscopic hysterectomy (malignant)	Prospective	Multiple	101	1	0	63	26	Severe cardiorespiratory disease, metastases	24^b^
2006	Kafy et al. [37]	Canada	Abdominal hysterectomy (benign)	Retrospective	Single	1,349	2	1	47	26	Peripartum or malignant hysterectomy	–
			Laparoscopic assisted vaginal hysterectomy (benign)	Retrospective	Single	223	2	0	47	28	Peripartum or malignant hysterectomy	–
2006	Mahdavi et al. [38]	USA	Laparoscopic hysterectomy (mixed)	Retrospective	Single	159	1	0	54	26	–	–
2006	Roman [39]	New Zealand	Laparoscopic assisted vaginal hysterectomy (mixed)	Retrospective	Single	418	0	0	46	–	–	–
2006	Sharon et al. [40]	Israel	Laparoscopic hysterectomy (mixed)	Retrospective	Single	480	1	1	50	–	Uterus size >16 weeks, PID	–
2007	Johnston et al. [41]	Australia	Laparoscopic assisted vaginal hysterectomy (benign)	Prospective	Multiple	69	2	0	–	–	–	–
2007	Karaman et al. [42]	Turkey	Laparoscopic assisted vaginal hysterectomy (benign)	Prospective	Multiple	542	0	0	–	–	Cancer, suspicious adnexal mass, uterine size >16 weeks	–
			Laparoscopic hysterectomy (benign)	Prospective	Multiple	552	0	0	–	–	Cancer, suspicious adnexal mass, uterine size >16 weeks	–
2007	Leonard et al. [43]	France	Laparoscopic hysterectomy (benign)	Retrospective	Single	1,300	–	4	48	23	POP, SUI	–
2007	Nawaz et al. [44]	Pakistan	Abdominal hysterectomy (benign)	Retrospective	Single	3,910	21	14	–	–	–	–
			Caesarean section	Retrospective	Single	12,567	31	2	–	–	–	–
			Vaginal hysterectomy (benign)	Retrospective	Single	481	9	2	–	–	–	–
2007	Ng and Chern [45]	Singapore	Laparoscopic hysterectomy (mixed)	Retrospective	Single	503	2	1	47	19	–	–
2007	O'Hanlan et al. [46]	USA	Laparoscopic hysterectomy (mixed)	Retrospective	Multiple	830	13	10	50	28	–	–
2007	Soong et al. [47]	Taiwan	Laparoscopic assisted vaginal hysterectomy (benign)	Retrospective	Single	7,725	30	8	–	–	–	–
2007	Tian et al. [48]	Taiwan	Laparoscopic assisted vaginal hysterectomy (mixed)	Retrospective	Single	2,174	10	2	–	–	Simultaneous diagnostic procedures	–
2007	Xu et al. [49]	China	Laparoscopic hysterectomy (malignant)	Retrospective	Single	317	9	5	–	–	–	6^b^
2008	Chen et al. [50]	China	Laparoscopic hysterectomy (malignant)	Retrospective	Single	290	5	1	43	–	–	46^a^
2008	Kyung et al. [51]	South Korea	Laparoscopic assisted vaginal hysterectomy (mixed)	Retrospective	Single	1,178	15	1	–	–	–	–
2009	Ark et al. [52]	Turkey	Laparoscopic assisted vaginal hysterectomy (benign)	Retrospective	Single	367	1	0	51	32	Cancer, prior surgery, <2 finger vaginal width, POP	–
2009	Chopin et al. [53]	France	Laparoscopic hysterectomy (benign)	Retrospective	Single	1,460	14	3	48	24	Cancer, POP, SUI, missing data	–
2009	Donnez et al. [54]	Belgium	Abdominal hysterectomy (benign)	Retrospective	Single	409	3	0	–	–	–	24^b^
			Vaginal hysterectomy (benign)	Retrospective	Single	906	4	3	–	–	–	24^c^
2009	Ibeanu et al. [55]	USA	Abdominal hysterectomy (benign)	Prospective	Multiple	544	12	9	–	–	Cancer, prior hysterectomy	–
			Laparoscopic assisted vaginal hysterectomy (benign)	Prospective	Multiple	61	2	0	–	–	Cancer, prior hysterectomy	–
2009	Juillard et al. [56]	USA	Abdominal hysterectomy (benign)	Retrospective	Multiple	172,344	1,239	19	46	–	–	–
2009	Lafay Pillet et al. [57]	France	Laparoscopic hysterectomy (benign)	Retrospective	Single	1,501	15	5	48	24	Subtotal hysterectomy, cancer	–
2009	Lee et al. [58]	Hong Kong	Laparoscopic assisted vaginal hysterectomy (benign)	Retrospective	Single	512	3	0	45	–	Cancer, uterine size >18 weeks	1.5^b^
2009	Rahman et al. [59]	Saudi Arabia	Caesarean section	Retrospective	Single	7,708	34	0	29	30	Simultaneous hysterectomy	1.5^b^
2009	Yan et al. [60]	China	Laparoscopic hysterectomy (malignant)	Retrospective	Single	117	6	1	41	–	–	–
2010	Gungorduk et al. [61]	Turkey	Caesarean section	Retrospective	Single	56,799	76	–	–	–	Simultaneous hysterectomy	1.5^b^
2010	Wang et al. [62]	Australia	Laparoscopic hysterectomy (benign)	Retrospective	Multiple	574	12	2	–	–	Missing data	1.5^b^
2010	Wright et al. [63]	USA	Abdominal hysterectomy (mixed)	Retrospective	Multiple	578,179	6,178	711	–	–	Cancer	–
			Peripartum hysterectomy	Retrospective	Multiple	4,967	458	33	–	–	Cancer	–
2011	Anpalagan et al. [64]	Australia	Abdominal hysterectomy (benign)	Retrospective	Multiple	87	0	0	–	–	–	1.5^b^
			Laparoscopic hysterectomy (benign)	Retrospective	Multiple	991	14	0	–	–	–	1.5^b^
2011	Brummer et al. [65]	Finland	Abdominal hysterectomy (benign)	Prospective	Multiple	1,255	11	4	–	–	Nil	–
			Laparoscopic hysterectomy (benign)	Prospective	Multiple	1,679	17	5	–	–	Nil	–
			Vaginal hysterectomy (benign)	Prospective	Multiple	2,345	14	1	–	–	Nil	–
2011	Doganay et al. [66]	Turkey	Abdominal hysterectomy (benign)	Retrospective	Single	4,398	30	8	54	–	Cancer, other procedures, clotting disorders	–
			Vaginal hysterectomy (benign)	Retrospective	Single	1,944	7	2	54	–	Cancer, other procedures, clotting disorders	–
2011	Jung and Lee [67]	South Korea	Laparoscopic assisted vaginal hysterectomy (benign)	Retrospective	Single	458	0	0	49	25	Cancer	–
2011	Kavallaris et al. [68]	Germany	Laparoscopic assisted vaginal hysterectomy (benign)	Retrospective	Multiple	1,255	11	2	46	27	–	–
2011	Song et al. [69]	South Korea	Laparoscopic assisted vaginal hysterectomy (mixed)	Retrospective	Single	2,012	26	1	45	24	–	80^a^
2012	Al-Shahrani et al. [70]	Saudi Arabia	Caesarean section	Retrospective	Single	10,765	24	–	–	–	–	–
2012	Cho et al. [71]	South Korea	Vaginal hysterectomy (benign)	Retrospective	Single	686	3	0	45	24	Cancer, ovary >5 cm	0.25^b^
2012	Grosse-Drieling et al. [72] (71)	Germany	Laparoscopic hysterectomy (benign)	Retrospective	Single	1,584	4	1	46	25	Cancer	–
2012	Khan et al. [73]	Pakistan	Peripartum hysterectomy	Retrospective	Single	218	6	1	–	–	–	–
2012	Kobayashi et al. [74]	Japan	Laparoscopic hysterectomy (mixed)	Retrospective	Single	1,253	6	4	46	23	Nil	–
2012	Lee et al. [75]	South Korea	Abdominal hysterectomy (mixed)	Retrospective	Single	6,792	19	7	–	–	–	6^b^
			Laparoscopic assisted vaginal hysterectomy (mixed)	Retrospective	Single	2,891	8	7	–	–	–	6^b^
			Laparoscopic hysterectomy (mixed)	Retrospective	Single	1,625	0	0	–	–	–	6^b^
			Vaginal hysterectomy (mixed)	Retrospective	Single	5,182	16	5	–	–	–	6^b^
2012	Mueller et al. [76]	Germany	Laparoscopic hysterectomy (benign)	Retrospective	Single	567	4	1	48	26	Simultaneous POP surgery, cancer	–
2012	Rao et al. [77]	China	Caesarean section	Retrospective	Single	6,732	8	1	–	–	–	5^b^
2012	Sandberg et al. [78]	USA	Abdominal hysterectomy (mixed)	Retrospective	Single	644	5	0	–	–	Peripartum hysterectomy	–
			Laparoscopic hysterectomy (mixed)	Retrospective	Single	1,011	7	4	–	–	Peripartum hysterectomy	–
			Robot–assisted hysterectomy (mixed)	Retrospective	Single	77	0	1	–	–	Peripartum hysterectomy	–
			Vaginal hysterectomy (mixed)	Retrospective	Single	250	2	0	–	–	Peripartum hysterectomy	–
2012	Teerapong et al. [79]	Thailand	Laparoscopic assisted vaginal hysterectomy (benign)	Retrospective	Single	101	4	3	–	–	–	1.5^b^
2012	Tuuli et al. [80]	USA	Caesarean section	RCT	Single	258	0	0	27	–	Emergency LSCS, prior surgery, gestation <32 weeks	1^b^
2013	Choi et al. [81]	South Korea	Laparoscopic assisted vaginal hysterectomy (benign)	Retrospective	Single	250	3	0	49	23	Cancer	–
2013	Mäkinen et al. [82]	Finland	Abdominal hysterectomy (benign)	Retrospective	Multiple	7,130	38	13	49	26	–	–
			Laparoscopic hysterectomy (benign)	Retrospective	Multiple	4,113	47	31	48	25	–	–
			Vaginal hysterectomy (benign)	Retrospective	Multiple	4,146	16	1	57	26	–	–
2013	Sheth [83]	India	Vaginal hysterectomy (benign)	Retrospective	Multiple	536	5	0	–	–	<2 prior LSCS, POP, adhesions, uterus >20-week size, tubo-ovarian pathological condition	–
2014	Dutta and Dutta [84]	India	Abdominal hysterectomy (mixed)	Prospective	Single	1,450	6	1	–	–	–	–
2014	Han et al. [85]	China	Laparoscopic hysterectomy (malignant)	Prospective	Single	176	0	6	45	–	–	–
2014	Nguyen et al. [86]	USA	Robot-assisted hysterectomy (mixed)	Retrospective	Multiple	229	0	0	58	33	Convert robot-assisted to laparotomy	1^b^
2014	Park and Nam [87]	South Korea	Laparoscopic hysterectomy (malignant)	Retrospective	Single	260	3	1	48	23	Nil	–
2014	Zia and Rafique [88]	Saudi Arabia	Caesarean section	Retrospective	Single	519	6	0	33	–	Placental adhesion disorders, <28 weeks gestation	–
2015	Garabedian et al. [89]	France	Laparoscopic hysterectomy (malignant)	Retrospective	Single	170	3	2	47	26	–	48^a^
2015	Kaplanoglu et al. [90]	Turkey	Caesarean section	Retrospective	Single	2,460	28	0	30	–	Syrian refugees, lack of follow-up	1.5^b^
2015	Tan-Kim et al. [91]	USA	Abdominal hysterectomy (benign)	Retrospective	Single	140	5	2	–	–	–	42^c^
2016	Dolanbay et al. [92]	Turkey	Laparoscopic assisted vaginal hysterectomy (benign)	Retrospective	Single	184	2	0	46	–	–	–
2016	Kang et al. [93]	South Korea	Laparoscopic hysterectomy (mixed)	Retrospective	Single	746	6	3	46	–	Advanced malignancy	1^b^
2016	Tinelli et al. [94]	Italy	Laparoscopic hysterectomy (malignant)	Retrospective	Single	110	3	4	62	37	Stage III or IV cancer, prior chemo- or radio-therapy, systemic infection, uterus >12-week size, significant cardiorespiratory disease, unclear follow-up	38^c^
2017	Clave and Clave [91]	France	Vaginal hysterectomy (benign)	Retrospective	Single	1,000	12	0	51	26	–	12^b^
2017	Lim et al. [96]	South Korea	Laparoscopic hysterectomy (mixed)	Retrospective	Single	482	1	0	49	24	Prior abdominal surgery, simultaneous surgery	–
2017	Satitniramai and Manonai [97]	Thailand	Abdominal hysterectomy (mixed)	Retrospective	Single	13,288	36	18	–	–	–	–
			Laparoscopic hysterectomy (mixed)	Retrospective	Single	2,131	4	6	–	–	–	–
2017	Singla et al. [98]	India	Peripartum hysterectomy	Retrospective	Single	194	13	0	–	–	–	–
2017	Yaman Tunc et al. [99]	Turkey	Caesarean section	Retrospective	Single	1,133	14	0	31	–	<24 weeks gestation, multiparous, prior surgery, stillbirth, missing data	–
2018	Benson et al. [100]	USA	Abdominal hysterectomy (benign)	Retrospective	Multiple	355,812	3,760	1,686	–	–	Cancer	–
			Laparoscopic hysterectomy (benign)	Retrospective	Multiple	31,389	830	158	–	–	Cancer	–
			Vaginal hysterectomy (benign)	Retrospective	Multiple	123,139	1,133	61	–	–	Cancer	–
2018	Blackwell et al. [3]	USA	Abdominal hysterectomy (mixed)	Retrospective	Multiple	99,693	–	953	–	–	Exenteration, prior hydro or ureteric stricture	12^d^
			Laparoscopic assisted vaginal hysterectomy (mixed)	Retrospective	Multiple	27,158	–	245	–	–	Exenteration, prior hydro or ureteric stricture	12^d^
			Laparoscopic hysterectomy (mixed)	Retrospective	Multiple	16,584	–	182	–	–	Exenteration, prior hydro or ureteric stricture	12^d^
			Peripartum hysterectomy	Retrospective	Multiple	1,528	–	21	–	–	Exenteration, prior hydro or ureteric stricture	12^d^
			Vaginal hysterectomy (mixed)	Retrospective	Multiple	45,002	–	100	–	–	Exenteration, prior hydro or ureteric stricture	12^d^
2018	Koroglu et al. [101]	Turkey	Laparoscopic hysterectomy (benign)	Retrospective	Single	504	2	1	49	32	Abscess, PID, POP, prior surgery, cancer, missing data	–
2018	Li et al. [102]	China	Abdominal hysterectomy (malignant)	Retrospective	Single	551	5	12	–	–	LUTS, loss to follow-up	42^a^
2018	Petersen et al. [103]	USA	Abdominal hysterectomy (mixed)	Retrospective	Single	940	4	5	50	33	–	3^b^
			Laparoscopic hysterectomy (mixed)	Retrospective	Single	782	3	4	50	33	–	3
			Robot-assisted hysterectomy (mixed)	Retrospective	Single	1,088	3	7	50	33	–	3^b^
			Vaginal hysterectomy (mixed)	Retrospective	Single	304	0	1	50	33	–	3^b^
2019	Inan et al. [101]	Turkey	Laparoscopic hysterectomy (benign)	Retrospective	Single	547	7	4	49	26	Cancer, other procedures	–
2019	Otkjaer et al. [105]	Denmark	Caesarean section	Retrospective	Multiple	4,039	12	0	31	24	Gestation <37 weeks, infant <2.5 kg, maternal comorbidity, emergency LSCS	–
2019	Sirota et al. [105]	USA	Vaginal hysterectomy (benign)	Retrospective	Single	452	13	3	57	–	POP	–
2019	Sondgeroth et al. [107]	USA	Caesarean section	Retrospective	Single	5,144	14	0	27	–	Non-singleton	–

*LSCS* lower section caesarean section, *LUTS* lower urinary tract symptoms, *PID* pelvic inflammatory disease, *POP* pelvic organ prolapse, *SUI* stress urinary incontinence

^a^Median

^b^Planned, not measured

^c^Mean

^d^Methodology searched for readmissions with bladder or ureteric injuries within 12 months of primary procedure

One study was a randomised controlled trial [80], whereas all the others were non-randomised observational studies. All but 12 studies were retrospective in nature. Mean or median age was reported by 67 of the 130 cohorts and ranged from 27 to 63 years. Similarly, mean or median body mass index was available for 42 cohorts, and varied from 19 to 37 kg/m^2^. Average American Society of Anaesthesiology score or Charlson Comorbidity Index were available for only 4 [34, 36, 95, 103] or 3 studies [3, 56, 87] respectively. Meta-analyses of bladder and ureteric injury rates are presented in Table 2 and Appendix 5.

**Table 2:** Meta-analyses by procedure sub-group

Procedure		Bladder				Ureteric			
	Studies identified	Studies reporting bladder injury rate	Patients (*n*)	Weighted pooled mean injury rates; events per 100,000 procedures	95% CI; events per 100,000 procedures	Studies reporting ureteric injury rate	Patients (*n*)	Weighted pooled mean injury rates; events per 100,000 procedures	95% CI; events per 100,000 procedures
Caesarean section	15	15	144,558	267	190–343	11	62,187	9	0–18
Hysterectomy									
Open abdominal (benign)	15	15	550,784	641	445–837	15	550,784	255	6–446
Open abdominal (any histology)	9	7	604,058	473	46–900	9	707,492	261	33–489
Open abdominal (malignant)	4	4	1,711	614	0–1,302	4	1,711	577	0–1,191
Open abdominal (peripartum)	4	3	5,379	6,279	1,731–10,826	4	6,907	666	178–1,153
Vaginal (benign)	15	15	144,856	878	635–1,120	15	144,856	39	23–55
Vaginal (any histology)	6	4	6,109	295	156–435	6	52,492	122	5–239
Laparoscopic (benign)	15	14	48,814	997	401–1,594	15	50,114	262	126–399
Laparoscopic (any histology)	13	11	10,002	375	173–577	13	27,022	417	127–707
Laparoscopic–(malignant)	8	8	1,541	1,553	610–2,496	8	1,541	814	222–1,406
Laparoscopic–assisted vaginal (benign)	14	14	12,077	445	190–699	14	12,077	87	26–147
Laparoscopic assisted vaginal (any histology)	9	8	13,530	506	248–764	9	40,688	186	0–415
Robot-assisted (any histology)	3	3	1,394	212	0–486	3	1,394	398	0–939

*CI* confidence interval,

### Caesarean section

As >15 caesarean section cohorts were identified, the largest 15 were selected, representing 144,816 women [22, 27, 30, 35, 44, 59, 61, 70, 77, 80, 88, 90, 99, 105, 107]. In total, 312 bladder and 7 ureteric injuries were reported. Weighted pooled mean injury rates were 267 and 9 events per 100,000 procedures respectively.

### Open abdominal hysterectomy (benign histology)

The 15 largest cohorts were selected, comprising 550,784 women [13, 14, 24, 32, 37, 44, 54–56, 64–66, 82, 91, 100]. Cumulatively, 5,138 bladder and 1,768 ureteric injuries were detected. Weighted pooled mean injury rates were 641 and 255 events per 100,000 cases respectively.

### Open abdominal hysterectomy (malignant histology)

Four cohorts were found, representing 1,711 women [24, 28, 31, 102]. Together, 15 bladder and 15 ureteric injuries were described. Weighted pooled mean injury rates were 614 and 577 events per 100,000 cases respectively.

### Open abdominal hysterectomy (any histology)

Nine cohorts were located, totalling 707,492 women [3, 15, 16, 63, 75, 78, 84, 97, 103]. Summatively, 6,252 bladder and 1,712 ureteric injuries were reported. Weighted pooled mean injury rates were 473 and 261 events per 100,000 cases respectively.

### Open abdominal hysterectomy (peripartum)

Amongst 6,907 women in four cohorts, 477 bladder and 55 ureteric injuries were reported in total [3, 63, 73, 98]. Weighted pooled mean injury rates were 6,279 and 666 events per 100,000 cases, respectively.

### Vaginal hysterectomy (benign histology)

The largest 15 cohorts were selected, comprising 144,856 women [14, 17, 21, 24, 33, 44, 54, 65, 66, 71, 82, 83, 95, 100, 106]. In aggregate, 1,323 bladder and 75 ureteric injuries occurred. Weighted pooled mean injury rates were 878 and 39 events per 100,000 cases respectively.

### Vaginal hysterectomy (any histology)

From the six identified cohorts representing 52,492 women, 20 bladder and 100 ureteric injuries were noted [3, 15, 16, 75, 78, 103]. Weighted pooled mean injury rates were 295 and 122 events per 100,000 cases respectively.

### Laparoscopic hysterectomy (benign histology)

Within the 15 largest cohorts constituting 50,114 women, 988 bladder and 222 ureteric injuries were observed [20, 34, 42, 43, 53, 57, 62, 64, 65, 72, 76, 82, 100, 101, 104]. Weighted pooled mean injury rates were 997 and 262 events per 100,000 cases respectively.

### Laparoscopic hysterectomy (malignant histology)

Eight cohorts were found, comprising 1,541 women [36, 49, 50, 60, 85, 87, 89, 94]. Thirty bladder and 20 ureteric injuries were reported. Weighted pooled mean injury rates were 1,553 and 814 events per 100,000 cases respectively.

### Laparoscopic hysterectomy (any histology)

Thirteen cohorts incorporating 27,022 women were identified, with 44 bladder and 221 ureteric injuries recorded [3, 19, 38, 40, 45, 46, 74, 75, 78, 93, 96, 97, 103]. Weighted pooled mean injury rates were 375 and 417 events per 100,000 cases respectively.

### Laparoscopically assisted vaginal hysterectomy (benign)

Within 14 cohorts totalling 12,077 women, 65 bladder and 13 ureteric injuries were detailed [18, 29, 37, 41, 42, 47, 52, 55, 58, 67, 68, 79, 81, 92]. Weighted pooled mean injury rates were 445 and 87 events per 100,000 cases respectively.

### Laparoscopically assisted vaginal hysterectomy (any histology)

Nine cohorts were identified, representing 40,688 women. A total of 81 bladder and 260 ureteric injuries were recorded [3, 23, 25, 26, 39, 48, 51, 69, 75]. Weighted pooled mean injury rates were 506 and 186 events per 100,000 cases respectively.

### Robot-assisted hysterectomy (any histology)

Three cohorts were found, constituting 1,394 women [78, 86, 103]. Together, 3 bladder and 8 ureteric injuries were noted. Weighted pooled mean injury rates were 212 and 398 events per 100,000 cases respectively.

### Risk factors for urological injury

Fifty-two studies identified one or more risk factors for bladder or ureteric injury (Appendix 6). In descending order of frequency, the most common elements were surgeon inexperience or low volume (18 studies), prior caesarean section (17), other previous pelvic surgery (14), adhesions (10), large uterus or tumour (10), endometriosis (8), cancer (5), radiotherapy (5), above average haemorrhage (5), low or high body mass index (5), placental adhesion disorder (4), concomitant surgery (4) and emergency procedure (2). Note that “surgeon inexperience or low volume” was self-defined by each study, and included undefined [28, 34, 49, 50, 59, 62, 77, 78, 93, 96], a surgical trainee [97], a consultant with <8 years’ experience [41] or having been primary operator for a given hysterectomy approach for fewer than 20 [23], 30 [40, 82], 50 [43, 87] or 100 cases [57].

### Preventing urological injury

Strategies to reduce the risk of bladder or ureteric injury during caesarean section or hysterectomy were promoted by 35 studies (Appendix 7). From most to least common, recommended measures included improved anatomical knowledge (12 studies), strong uterine traction (12), careful dissection generally (9), prophylactic identification of ureters (8), bladder distension with fluid to clarify planes (7), avoiding diathermy near ureters (6), actively dissecting (as opposed to blunt traction) the bladder away from uterus (3), urethral catheterisation at the start of the case (3), prophylactic ureteric catheters/stents (3) and shielding the bladder with retractors (2).

### Assessment of bias

The Newcastle–Ottawa Quality Assessment Scale suggested that the risk of bias was intermediate (46 studies) or high (35 studies) for most of the 96 works identified (Appendix 8). Key methodological and governance information was frequently absent, including typical post-operative follow-up (missing in 70% of studies), financial disclosure (missing in 73%), conflict of interest (missing in 53%) and ethics approval (missing or explicitly not present in 52%). Eighty four of the 96 studies were retrospective and therefore at an increased risk of selection bias. Publication bias was not assessed, given the studies’ heterogenous methodology and array of procedure sub-types.

## Discussion

To our knowledge, this represents the largest systematic review to date of urological injury during major obstetric and gynaecological surgery. For clinicians performing caesarean section or hysterectomy, these findings may aid their daily practice in three ways. First, the pooled mean injury incidence may aid in counselling patients on the risk of bladder or ureteric injury for caesarean section or their specific surgical approach to hysterectomy. Our findings suggest that rates of bladder and ureteric injury are low for caesarean section and most approaches to hysterectomy. It is clear that both bladder and ureteric injury risk are highest during peripartum and laparoscopic radical hysterectomy. We believe that for peripartum hysterectomy this relates to poor visibility from haemorrhage from the gravid uterus and to placental invasion disorders, which may distort anatomical planes and invade the bladder, and for malignant hysterectomy to the desire to dissect widely to achieve negative margins and challenges from tumour infiltration. Analyses between tumour stage and injury incidence were not performed. Second, the collated risk factors can aid in the pre-operative assessment of a specific patient’s risk of urological injury. This information may be used to involve a senior surgeon, prophylactically identify and safeguard the ureters, refer to a tertiary centre or employ other cautionary steps. Third, the advocated preventative strategies can be both incorporated into routine practice and utilised more intensively in settings of known increased risk, as identified above.

This review’s relevance is underlined by the ongoing high volume and changing surgical approaches of major obstetric and gynaecological surgery. Globally, there is a trend towards more caesarean sections [108], with >30 million performed annually, comprising >20% of all births. Well over 1 million hysterectomies are performed annually [109], although the incidence is slowly declining in most [110, 111] but not all nations [112]. Simultaneously, as with other specialties, the approach to hysterectomy continues to shift towards minimally invasive means. Twenty years ago, abdominal (open) hysterectomy was the most common technique in both developed and developing nations [82, 110, 111, 113, 114]. From this baseline, minimally invasive approaches are now the most common in developed nations. Robot-assisted hysterectomy is the most common approach in the United States of America [115], laparoscopic hysterectomy predominates in Australia [114], Denmark [111] and Taiwan [113], whereas the vaginal approach is customary in Austria [116]. In developing nations, abdominal hysterectomy remains the norm [117].

Some authors recommended preventative strategies of routine cystoscopy (often with intravenous indigo carmine [3, 15, 23, 32, 40, 41, 43, 45, 51, 55, 93]) or prophylactic placement of ureteric stents. However, the evidence suggests that neither of these might be sufficiently sensitive or cost effective. Intra-operative cystoscopy seems a logical precaution, allowing prompt inspection for haematuria, intact urothelium, ureteric jets and blood from the ureteric orifices. However, where practised, cystoscopy is diagnostic and not preventative, being performed at the end of the gynaecological procedure to detect an injury that has already occurred. Furthermore, cystoscopy has low sensitivity for both bladder [91] and ureteric [19, 78, 91] injuries. Regarding prophylactic stents, randomised controlled trials of their use in gynaecological procedures have given mixed results regarding reduced rates of ureteric injury [118, 119]. However, ureteric stent use may reduce diagnostic delay and post-operative morbidity [120].

Many clinicians may not appreciate the significant risk of death in women with ureteric injuries. Although most bladder injuries are diagnosed intra-operatively, most ureteric injuries are detected post-operatively, with a typical diagnostic delay of 10–14 days [3, 40, 47, 65]. A study of >200,000 women undergoing hysterectomy found that compared with patients with no ureteric injury, patients with a delayed diagnosis of ureteric injury have significantly lower 1-year overall survival (99.7% vs 91.7%) [3]. The reasons for the reduced survival were not assessed. This 1 in 12 risk of death at 1 year, akin to stage IIIC colorectal cancer [121], is a terrifying prospect for these patients, who are predominantly women aged 30–50 years undergoing hysterectomy for a benign indication [110]. Separate to death is the inconvenience and morbidity of further interventions. Although some ureteric injuries may be managed by minimally invasive means such as ureteric stent insertion, most will require formal repair via ureteric reimplantation (neoureterocystostomy) [40, 46, 47, 122]. Some selected cases will require additional measures such as a psoas hitch, Boari flap, uretero-ureterostomy or bowel interposition [65]. Some require up to six further procedures [91].

The leading risk factor for urological injury was surgeon inexperience or low volume, identified by 18 studies [23, 28, 34, 40, 41, 43, 49, 50, 57, 59, 62, 77, 78, 82, 87, 93, 96, 97]. A strong demonstration of this is Mäkinen et al.’s Finnish study of >10,000 hysterectomies. This found that, compared with surgeons who had performed ≤30 laparoscopic hysterectomies, those with experience of >30 procedures had a significantly lower rate of injury to the bladder (2.2% vs 0.8%) or ureter (2.0% vs 0.5%) [123]. Gynaecological trainees and consultants early in their learning curve may benefit their patients by increasing their supervision during this time, as well as incorporating the most commonly advocated preventative strategies of improving their anatomical knowledge, and intra-operative techniques of strong uterine traction and careful dissection generally.

This review’s strengths are its comprehensive curation and critique of the literature. It is limited by the lack of randomised trials and the heterogeneous methodology of the studies included. Non-randomised studies are more prone to bias; thus, these results should be interpreted with caution. However, as pointed out by some of the identified studies [82], national registry-based observational studies may better reflect clinical reality in the hands of the “average” gynaecological surgeon than randomised controlled trials. Exclusion of non-English publications is another limitation. Additionally, this review’s inclusion criteria sought to balance broad inclusion with manageable data collection. The decision to include the 15 largest studies for each surgical approach, regardless of whether or not they observed any bladder or ureteric complications, may have reduced the number of studies for which sub-group meta-analysis was possible. Furthermore, the small number of eligible works identified for open abdominal hysterectomy for malignant histology (4 studies), vaginal hysterectomy for any histology (6 studies) and robot-assisted hysterectomy for any histology (3 studies) limits confidence in the findings for these sub-groups.

Many studies may have had inadequate follow-up to detect ureteric injuries. Sixty-eight of the 97 studies included did not detail their post-operative follow-up (Table 1). Of those that did, 19 out of 29 studies stated only their planned consultations, without measuring whether these occurred or not [3, 25, 36, 49, 54, 58, 59, 61, 62, 64, 71, 75, 77, 79, 80, 86, 90, 93, 95, 103]. As highlighted by Wang et al., “both short- and long-term follow up are required because complications may occur greater than four weeks after the initial surgery” [62]. Hence, this opaque provision of aftercare limits certainty that all events have been captured, and pooled complication rates may be higher than our findings suggest.

This study’s methodology confined its scope to bladder and ureteric injury. Caesarean section and hysterectomy may cause other urological complications, such as transient urinary retention, nerve injury causing atonic bladder [89], vesico-vaginal fistula or uretero-vaginal fistula [49]. These were not assessed by this review.

## Conclusion

Caesarean section and most types of hysterectomy carry low rates of bladder and ureteric injury. Surgeon inexperience represents the leading risk factor for iatrogenic injury. Improved anatomical knowledge is the most commonly suggested preventative strategy. Obstetricians and gynaecologists should counsel the patient for her individual risk of injury, prospectively establish risk factors and implement preventative strategies to minimise risk.

## Supplementary information

ESM 1 (PDF 4.42 mb)
