# Supplementary material for: Systematic review of urological injury during caesarean section and hysterectomy
Source: Int Urogynecol J. 2022 Oct 17;34(2):371–89. doi: 10.1007/s00192-022-05339-7 (PMC9870963; doi:10.1007/s00192-022-05339-7)
Supplement: Supplementary file 1 — (PDF 4.42 mb) [file 192_2022_5339_MOESM1_ESM.pdf]

Appendix 1. PRISMA 2020 Checklist

Section and Topic	Item #	Checklist item	Location where item is reported
**TITLE**			
Title	1	Identify the report as a systematic review.	Title page
**ABSTRACT**			
Abstract	2	See the PRISMA 2020 for Abstracts checklist.	Abstract
**INTRODUCTION**			
Rationale	3	Describe the rationale for the review in the context of existing knowledge.	5
Objectives	4	Provide an explicit statement of the objective(s) or question(s) the review addresses.	5
**METHODS**			
Eligibility criteria	5	Specify the inclusion and exclusion criteria for the review and how studies were grouped for the syntheses.	6
Information sources	6	Specify all databases, registers, websites, organisations, reference lists and other sources searched or consulted to identify studies. Specify the date when each source was last searched or consulted.	6
Search strategy	7	Present the full search strategies for all databases, registers and websites, including any filters and limits used.	5
Selection process	8	Specify the methods used to decide whether a study met the inclusion criteria of the review, including how many reviewers screened each record and each report retrieved, whether they worked independently, and if applicable, details of automation tools used in the process.	6
Data collection process	9	Specify the methods used to collect data from reports, including how many reviewers collected data from each report, whether they worked independently, any processes for obtaining or confirming data from study investigators, and if applicable, details of automation tools used in the process.	6
Data items	10a	List and define all outcomes for which data were sought. Specify whether all results that were compatible with each outcome domain in each study were sought (e.g. for all measures, time points, analyses), and if not, the methods used to decide which results to collect.	7
	10b	List and define all other variables for which data were sought (e.g. participant and intervention characteristics, funding sources). Describe any assumptions made about any missing or unclear information.	6 & Appendix 2
Study risk of bias assessment	11	Specify the methods used to assess risk of bias in the included studies, including details of the tool(s) used, how many reviewers assessed each study and whether they worked independently, and if applicable, details of automation tools used in the process.	7
Effect measures	12	Specify for each outcome the effect measure(s) (e.g. risk ratio, mean difference) used in the synthesis or presentation of results.	7
Synthesis methods	13a	Describe the processes used to decide which studies were eligible for each synthesis (e.g. tabulating the study intervention characteristics and comparing against the planned groups for each synthesis (item #5)).	6
	13b	Describe any methods required to prepare the data for presentation or synthesis, such as handling of missing summary statistics, or data conversions.	6
	13c	Describe any methods used to tabulate or visually display results of individual studies and syntheses.	6
	13d	Describe any methods used to synthesize results and provide a rationale for the choice(s). If meta-analysis was performed, describe the model(s), method(s) to identify the presence and extent of statistical heterogeneity, and software package(s) used.	6
	13e	Describe any methods used to explore possible causes of heterogeneity among study results (e.g. subgroup analysis, meta-regression).	6
	13f	Describe any sensitivity analyses conducted to assess robustness of the synthesized results.	n/a
Reporting bias assessment	14	Describe any methods used to assess risk of bias due to missing results in a synthesis (arising from reporting biases).	7 & Appendix 3
Certainty assessment	15	Describe any methods used to assess certainty (or confidence) in the body of evidence for an outcome.	n/a

PRISMA 2020 Checklist

Section and Topic	Item #	Checklist item	Location where item is reported
**RESULTS**			
Study selection	16a	Describe the results of the search and selection process, from the number of records identified in the search to the number of studies included in the review, ideally using a flow diagram.	8 & Figure 1
	16b	Cite studies that might appear to meet the inclusion criteria, but which were excluded, and explain why they were excluded.	8 & Appendix 4
Study characteristics	17	Cite each included study and present its characteristics.	Table 2
Risk of bias in studies	18	Present assessments of risk of bias for each included study.	Appendix 8
Results of individual studies	19	For all outcomes, present, for each study: (a) summary statistics for each group (where appropriate) and (b) an effect estimate and its precision (e.g. confidence/credible interval), ideally using structured tables or plots.	
Results of syntheses	20a	For each synthesis, briefly summarise the characteristics and risk of bias among contributing studies.	p.8-11
	20b	Present results of all statistical syntheses conducted. If meta-analysis was done, present for each the summary estimate and its precision (e.g. confidence/credible interval) and measures of statistical heterogeneity. If comparing groups, describe the direction of the effect.	p.8-11 Appendix 5
	20c	Present results of all investigations of possible causes of heterogeneity among study results.	
	20d	Present results of all sensitivity analyses conducted to assess the robustness of the synthesized results.	n/a
Reporting biases	21	Present assessments of risk of bias due to missing results (arising from reporting biases) for each synthesis assessed.	
Certainty of evidence	22	Present assessments of certainty (or confidence) in the body of evidence for each outcome assessed.	n/a
**DISCUSSION**			
Discussion	23a	Provide a general interpretation of the results in the context of other evidence.	13
	23b	Discuss any limitations of the evidence included in the review.	16
	23c	Discuss any limitations of the review processes used.	16
	23d	Discuss implications of the results for practice, policy, and future research.	15
**OTHER INFORMATION**			
Registration and protocol	24a	Provide registration information for the review, including register name and registration number, or state that the review was not registered.	6
	24b	Indicate where the review protocol can be accessed, or state that a protocol was not prepared.	6
	24c	Describe and explain any amendments to information provided at registration or in the protocol.	6
Support	25	Describe sources of financial or non-financial support for the review, and the role of the funders or sponsors in the review.	18
Competing interests	26	Declare any competing interests of review authors.	18
Availability of data, code and other materials	27	Report which of the following are publicly available and where they can be found: template data collection forms; data extracted from included studies; data used for all analyses; analytic code; any other materials used in the review.	n/a

From: Page MJ, McKenzie JE, Bossuyt PM, Boutron I, Hoffmann TC, Mulrow CD, et al. The PRISMA 2020 statement: an updated guideline for reporting systematic reviews. BMJ 2021;372:n71. doi: 10.1136/bmj.n71

For more information, visit: http://www.prisma-statement.org/

Appendix 2. Data extraction pro-forma

**Reviewer:**	one/ two	**Data extraction:**	1st/ 2nd
**Study year:**		**1st author:**	
**Nation:**			

Single centre: yes/ no

Design: Retrospective/ prospective

**Surgery type:**	
**Patients excluded:**	

	Numbers
**Total patients**	
**Bladder injuries**	
**Ureteric injuries**	

**Risk factors for injury**	
**Strategies to prevent injury**	

**Period (mo)**			
**Funding**		**Ethics approval**	
**Conflict of interest**			

**Comments**	

Appendix 3. Reviewer guidelines for completing the Newcastle-Ottawa Quality Assessment Scale

1. SELECTION

Representativeness of exposed cohort ★

Must enrol a cohort of consecutive adult patients, without restriction by age or comorbidity, undergoing hysterectomy or caesarean section. Cohorts may restrict themselves by hysterectomy indication, such as exclusively benign or malignant. Cohorts must clarify their exclusion criteria.

Selection of non-exposed cohort ★

Not applicable

Ascertainment of exposure ★

Scoring studies will reference hospital records or a prospective database.

Demonstration outcome of interest not present at start of study ★

Not applicable.

2. COMPARABILITY

Comparability of cohorts on the basis of the design or analysis ★★

One star given if the mean or median age of each cohort is given. A second star is given if other demographic data are given, such as American Society of Anaesthesiologist score, comorbidity score or body mass index.

3. OUTCOME

Assessment of outcome ★

Scoring studies will reference hospital records or a prospective database.

Was follow-up long enough for outcomes to occur? ★

Studies must detail a reasonable method for capture of late (post discharge) diagnosis bladder or ureteric injuries.

Adequacy of follow up of cohorts ★

Studies must detail that a reasonable method for capture of sequelae actually occurred.

Appendix 4. Studies retrieved for full text review.

1. Conde-Agudelo A. Intrafascial abdominal hysterectomy: outcomes and complications of 867 operations. Int J Gynaecol Obstet. 2000;68(3):233-9.
2. Leng J, Lang J, Huang R, Liu Z, Sun D. Complications in laparoscopic gynecologic surgery. Chin Med Sci J. 2000;15(4):222-6.
3. O'Shea RT, Gordon SJ, Seman EI, Verco CJ. Total laparoscopic tube hysterectomy: A safer option? Gynaecological Endoscopy. 2000;9(5):285-91.
4. Cosson M, Lambaudie E, Boukerrou M, Querleu D, Crepin G. Vaginal, laparoscopic, or abdominal hysterectomies for benign disorders: immediate and early postoperative complications. Eur J Obstet Gynecol Reprod Biol. 2001;98(2):231-6.
5. Leanza F, Bianca G, Cinquerrui G, Caschetto S. Lesions of the urinary organs during abdominal and vaginal hysterectomy. Urogynaecologia International Journal. 2001;15(2):25-31.
6. Liapis A, Bakas P, Giannopoulos V, Creatsas G. Ureteral injuries during gynecological surgery. Int Urogynecol J Pelvic Floor Dysfunct. 2001;12(6):391-3; discussion 4.
7. Makinen J, Johansson J, Tomas C, Tomas E, Heinonen PK, Laatikainen T, et al. Morbidity of 10 110 hysterectomies by type of approach. Hum Reprod. 2001;16(7):1473-8.
8. Mathevet P, Valencia P, Cousin C, Mellier G, Dargent D. Operative injuries during vaginal hysterectomy. Eur J Obstet Gynecol Reprod Biol. 2001;97(1):71-5.
9. Milad MP, Morrison K, Sokol A, Miller D, Kirkpatrick L. A comparison of laparoscopic supracervical hysterectomy vs laparoscopically assisted vaginal hysterectomy. Surg Endosc. 2001;15(3):286-8.
10. Seman EI, O'Shea RT, Gordon S, Miller J. Routine cystoscopy after laparoscopically assisted hysterectomy: What's the point? Gynaecological Endoscopy. 2001;10(4):253-6.
11. Wang PH, Lee WL, Yuan CC, Chao HT, Liu WM, Yu KJ, et al. Major complications of operative and diagnostic laparoscopy for gynecologic disease. J Am Assoc Gynecol Laparosc. 2001;8(1):68-73.
12. Wu MP, Lin YS, Chou CY. Major complications of operative gynecologic laparoscopy in southern Taiwan. J Am Assoc Gynecol Laparosc. 2001;8(1):61-7.
13. Wattiez A, Soriano D, Cohen SB, Nervo P, Canis M, Botchorishvili R, et al. The learning curve of total laparoscopic hysterectomy: comparative analysis of 1647 cases. J Am Assoc Gynecol Laparosc. 2002;9(3):339-45.
14. Boukerrou M, Lambaudie E, Collinet P, Crepin G, Cosson M. A history of cesareans is a risk factor in vaginal hysterectomies. Acta Obstetricia et Gynecologica Scandinavica. 2003;82(12):1135-9.
15. Khashoggi TY. Higher order multiple repeat cesarean sections: maternal and fetal outcome. Ann Saudi Med. 2003;23(5):278-82.
16. Miranda CS, Carvajal AR. Complications of operative gynecological laparoscopy. JSLS. 2003;7(1):53-8.
17. O'Hanlan KA, Lopez L, Dibble SL, Garnier AC, Huang GS, Leuchtenberger M. Total laparoscopic hysterectomy: body mass index and outcomes. Obstet Gynecol. 2003;102(6):1384-92.
18. Shen CC, Wu MP, Kung FT, Huang FJ, Hsieh CH, Lan KC, et al. Major complications associated with laparoscopic-assisted vaginal hysterectomy: ten-year experience. J Am Assoc Gynecol Laparosc. 2003;10(2):147-53.
19. Dorairajan G, Rani PR, Habeebullah S, Dorairajan LN. Urological injuries during hysterectomies: a 6-year review. J Obstet Gynaecol Res. 2004;30(6):430-5.
20. Horng SG, Huang KG, Lo TS, Soong YK. Bladder injury after LAVH: a prospective, randomized comparison of vaginal and laparoscopic approaches to colpotomy during LAVH. J Am Assoc Gynecol Laparosc. 2004;11(1):42-6.
21. Koh LW, Koh PH, Lin LC, Ng WJ, Wong E, Huang MH. A simple procedure for the prevention of ureteral injury in laparoscopic-assisted vaginal hysterectomy. J Am Assoc Gynecol Laparosc. 2004;11(2):167-9.
22. Li Z, Leng J, Lang J, Tang J. Vaginal hysterectomy for patients with moderately enlarged uterus of benign lesions. Chin Med Sci J. 2004;19(1):60-3.
23. Parkar RB, Thagana NG, Otieno D. Laparoscopic assisted vaginal hysterectomy for benign uterine pathology: is it time to change? East African medical journal. 2004;81(5):261-6.
24. Rashid M, Rashid RS. Higher order repeat caesarean sections: How safe are five or more? BJOG: An International Journal of Obstetrics and Gynaecology. 2004;111(10):1090-4.
25. Steed H, Rosen B, Murphy J, Laframboise S, De Petrillo D, Covens A. A comparison of laparascopic-assisted radical vaginal hysterectomy and radical abdominal hysterectomy in the treatment of cervical cancer. Gynecol Oncol. 2004;93(3):588-93.
26. Chang WC, Torng PL, Huang SC, Sheu BC, Hsu WC, Chen RJ, et al. Laparoscopic-assisted vaginal hysterectomy with uterine artery ligation through retrograde umbilical ligament tracking. Journal of Minimally Invasive Gynecology. 2005;12(4):336-42.
27. Phipps MG, Watabe B, Clemons JL, Weitzen S, Myers DL. Risk factors for bladder injury during cesarean delivery. Obstet Gynecol. 2005;105(1):156-60.
28. Tae Kim Y, Sung Yoon B, Hoon Kim S, Hoon Kim J, Wook Kim J, Won Park Y. The influence of time intervals between loop electrosurgical excision and subsequent hysterectomy on the morbidity of patients with cervical neoplasia. Gynecologic Oncology. 2005;96(2):500-3.
29. Vakili B, Chesson RR, Kyle BL, Shobeiri SA, Echols KT, Gist R, et al. The incidence of urinary tract injury during hysterectomy: a prospective analysis based on universal cystoscopy. Am J Obstet Gynecol. 2005;192(5):1599-604.
30. Akyol D, Esinler I, Guven S, Salman MC, Ayhan A. Vaginal hysterectomy: results and complications of 886 patients. J Obstet Gynaecol. 2006;26(8):777-81.
31. Bojahr B, Raatz D, Schonleber G, Abri C, Ohlinger R. Perioperative complication rate in 1706 patients after a standardized laparoscopic supracervical hysterectomy technique. J Minim Invasive Gynecol. 2006;13(3):183-9.
32. Dauleh W, Al Sakka M, Shahata M. Urinary tract injuries during caesarean section. Qatar Medical Journal. 2006;15(2):8-10.
33. Ghezzi F, Cromi A, Bergamini V, Uccella S, Beretta P, Franchi M, et al. Laparoscopic management of endometrial cancer in nonobese and obese women: A consecutive series. J Minim Invasive Gynecol. 2006;13(4):269-75.
34. Kafy S, Huang JY, Al-Sunaidi M, Wiener D, Tulandi T. Audit of morbidity and mortality rates of 1792 hysterectomies. J Minim Invasive Gynecol. 2006;13(1):55-9.
35. Mahdavi A, Peiretti M, Dennis S, Nezhat F. Comparison of laparoscopic hysterectomy morbidity for gynecologic, oncologic, and benign gynecologic conditions. JSLS. 2006;10(4):439-42.
36. Roman JD. Patient selection and surgical technique may reduce major complications of laparoscopic-assisted vaginal hysterectomy. Journal of Minimally Invasive Gynecology. 2006;13(4):306-10.
37. Sharon A, Auslander R, Brandes-Klein O, Alter Z, Kaufman Y, Lissak A. Cystoscopy after total or subtotal laparoscopic hysterectomy: The value of a routine procedure. Gynecological Surgery. 2006;3(2):122-7.
38. Sobande A, Eskandar M. Multiple repeat caesarean sections: complications and outcomes. J Obstet Gynaecol Can. 2006;28(3):193-7.
39. Jelovsek JE, Chiung C, Chen G, Roberts SL, Paraiso MF, Falcone T. Incidence of lower urinary tract injury at the time of total laparoscopic hysterectomy. JSLS : Journal of the Society of Laparoendoscopic Surgeons / Society of Laparoendoscopic Surgeons. 2007;11(4):422-7.
40. Johnston K, Rosen D, Cario G, Chou D, Carlton M, Cooper M, et al. Major complications arising from 1265 operative laparoscopic cases: a prospective review from a single center. J Minim Invasive Gynecol. 2007;14(3):339-44.
41. Karaman Y, Bingol B, Gunenc Z. Prevention of complications in laparoscopic hysterectomy: experience with 1120 cases performed by a single surgeon. J Minim Invasive Gynecol. 2007;14(1):78-84.
42. Leonard F, Fotso A, Borghese B, Chopin N, Foulot H, Chapron C. Ureteral complications from laparoscopic hysterectomy indicated for benign uterine pathologies: a 13-year experience in a continuous series of 1300 patients. Hum Reprod. 2007;22(7):2006-11.
43. Moodliar S, Moodley J, Esterhuizen TM. Complications associated with caesarean delivery in a setting with high HIV prevalence rates. Eur J Obstet Gynecol Reprod Biol. 2007;131(2):138-45.
44. Nawaz FH, Khan ZE, Rizvi J. Urinary tract injuries during obstetrics and gynaecological surgical procedures at the Aga Khan University Hospital Karachi, Pakistan: A 20-year review. Urologia Internationalis. 2007;78(2):106-11.
45. Ng CC, Chern BS. Total laparoscopic hysterectomy: a 5-year experience. Arch Gynecol Obstet. 2007;276(6):613-8.
46. O'Hanlan KA, Dibble SL, Garnier AC, Reuland ML. Total laparoscopic hysterectomy: technique and complications of 830 cases. JSLS. 2007;11(1):45-53.
47. Sahin Y. Vaginal hysterectomy and oophorectomy in women with 12-20 weeks' size uterus. Acta Obstet Gynecol Scand. 2007;86(11):1359-69.
48. Sizzi O, Rossetti A, Malzoni M, Minelli L, La Grotta F, Soranna L, et al. Italian multicenter study on complications of laparoscopic myomectomy. J Minim Invasive Gynecol. 2007;14(4):453-62.
49. Soong YK, Yu HT, Wang CJ, Lee CL, Huang HY. Urinary tract injury in laparoscopic-assisted vaginal hysterectomy. J Minim Invasive Gynecol. 2007;14(5):600-5.
50. Tian YF, Lin YS, Lu CL, Chia CC, Huang KF, Shih TY, et al. Major complications of operative gynecologic laparoscopy in southern Taiwan: a follow-up study. J Minim Invasive Gynecol. 2007;14(3):284-92.
51. Xu H, Chen Y, Li Y, Zhang Q, Wang D, Liang Z. Complications of laparoscopic radical hysterectomy and lymphadenectomy for invasive cervical cancer: experience based on 317 procedures. Surg Endosc. 2007;21(6):960-4.
52. Berretta R, Merisio C, Melpignano M, Rolla M, Ceccaroni M, A DEI, et al. Vaginal versus abdominal hysterectomy in endometrial cancer: a retrospective study in a selective population. Int J Gynecol Cancer. 2008;18(4):797-802.
53. Chen Y, Xu H, Li Y, Wang D, Li J, Yuan J, et al. The outcome of laparoscopic radical hysterectomy and lymphadenectomy for cervical cancer: a prospective analysis of 295 patients. Ann Surg Oncol. 2008;15(10):2847-55.
54. Erian J, Hassan M, Pachydakis A, Chandakas S, Wissa I, Hill N. Efficacy of laparoscopic subtotal hysterectomy in the management of menorrhagia: 400 consecutive cases. BJOG. 2008;115(6):742-8.
55. Jung SK, Huh CY. Ureteral injuries during classic intrafascial supracervical hysterectomy: an 11-year experience in 1163 patients. J Minim Invasive Gynecol. 2008;15(4):440-5.
56. Kriplani A, Garg P, Sharma M, Lal S, Agarwal N. A review of total laparoscopic hysterectomy using ligasure uterine artery-sealing device: AIIMS experience. Journal of Laparoendoscopic and Advanced Surgical Techniques. 2008;18(6):825-9.
57. Kyung MS, Choi JS, Lee JH, Jung US, Lee KW. Laparoscopic management of complications in gynecologic laparoscopic surgery: a 5-year experience in a single center. J Minim Invasive Gynecol. 2008;15(6):689-94.
58. Likic IS, Kadija S, Ladjevic NG, Stefanovic A, Jeremic K, Petkovic S, et al. Analysis of urologic complications after radical hysterectomy. Am J Obstet Gynecol. 2008;199(6):644 e1-3.
59. Ark C, Gungorduk K, Celebi I, Celikkol O. Experience with laparoscopic-assisted vaginal hysterectomy for the enlarged uterus. Arch Gynecol Obstet. 2009;280(3):425-30.
60. Chopin N, Malaret JM, Lafay-Pillet MC, Fotso A, Foulot H, Chapron C. Total laparoscopic hysterectomy for benign uterine pathologies: Obesity does not increase the risk of complications. Human Reproduction. 2009;24(12):3057-62.
61. Donnez O, Jadoul P, Squifflet J, Donnez J. A series of 3190 laparoscopic hysterectomies for benign disease from 1990 to 2006: evaluation of complications compared with vaginal and abdominal procedures. BJOG. 2009;116(4):492-500.
62. Ibeanu OA, Chesson RR, Echols KT, Nieves M, Busangu F, Nolan TE. Urinary tract injury during hysterectomy based on universal cystoscopy. Obstet Gynecol. 2009;113(1):6-10.
63. Juillard C, Lashoher A, Sewell CA, Uddin S, Griffith JG, Chang DC. A National Analysis of the Relationship Between Hospital Volume, Academic Center Status, and Surgical Outcomes for Abdominal Hysterectomy Done for Leiomyoma. Journal of the American College of Surgeons. 2009;208(4):599-606.
64. Khursheed F, Sirichand P, Jatoi N. Intraoperative complications encountered in patients with repeat cesarean section. Journal of the Liaquat University of Medical and Health Sciences. 2009;8(1):76-9.
65. Lafay Pillet MC, Leonard F, Chopin N, Malaret JM, Borghese B, Foulot H, et al. Incidence and risk factors of bladder injuries during laparoscopic hysterectomy indicated for benign uterine pathologies: a 14.5 years experience in a continuous series of 1501 procedures. Hum Reprod. 2009;24(4):842-9.
66. Lee ET, Wong FW, Lim CE. A modified technique of LAVH with the Biswas uterovaginal elevator. J Minim Invasive Gynecol. 2009;16(6):755-60.
67. Morsi H, Phillips G. Can centralised care of complex laparoscopic procedures prevent urinary tract injuries? Gynecological Surgery. 2009;6(3):237-43.
68. Mueller A, Renner SP, Haeberle L, Lermann J, Oppelt P, Beckmann MW, et al. Comparison of total laparoscopic hysterectomy (TLH) and laparoscopy-assisted supracervical hysterectomy (LASH) in women with uterine leiomyoma. Eur J Obstet Gynecol Reprod Biol. 2009;144(1):76-9.
69. O'Hanlan KA. Cystosufflation to prevent bladder injury. J Minim Invasive Gynecol. 2009;16(2):195-7.
70. Rahman MS, Gasem T, Al Suleiman SA, Al Jama FE, Burshaid S, Rahman J. Bladder injuries during cesarean section in a University Hospital: a 25-year review. Arch Gynecol Obstet. 2009;279(3):349-52.
71. Tan JJ, Tsaltas J, Hengrasmee P, Lawrence A, Najjar H. Evolution of the complications of laparoscopic hysterectomy after a decade: a follow up of the Monash experience. Aust N Z J Obstet Gynaecol. 2009;49(2):198-201.
72. Twijnstra ARH, Kianmanesh Rad NA, Smeets MJGH, Admiraal JF, Jansen FW. Twenty-first century laparoscopic hysterectomy: Should we not leave the vaginal step out? Gynecological Surgery. 2009;6(4):311-6.
73. Yan X, Li G, Shang H, Wang G, Chen L, Han Y. Complications of laparoscopic radical hysterectomy and pelvic lymphadenectomy-experience of 117 patients. International Journal of Gynecological Cancer. 2009;19(5):963-7.
74. Frankman EA, Wang L, Bunker CH, Lowder JL. Lower urinary tract injury in women in the United States, 1979-2006. Am J Obstet Gynecol. 2010;202(5):495 e1-5.
75. Gungorduk K, Asicioglu O, Celikkol O, Sudolmus S, Ark C. Iatrogenic bladder injuries during caesarean delivery: a case control study. J Obstet Gynaecol. 2010;30(7):667-70.
76. Mueller A, Thiel F, Lermann J, Oppelt P, Beckmann MW, Renner SP. Feasibility and safety of total laparoscopic hysterectomy (TLH) using the Hohl instrument in nonobese and obese women. Journal of Obstetrics and Gynaecology Research. 2010;36(1):159-64.
77. Salman MC, Usubutun A, Ozlu T, Boynukalin K, Yuce K. Obesity does not affect the number of retrieved lymph nodes and the rate of intraoperative complications in gynecologic cancers. Journal of Gynecologic Oncology. 2010;21(1):24-8.
78. Wang L, Merkur H, Hardas G, Soo S, Lujic S. Laparoscopic hysterectomy in the presence of previous caesarean section: a review of one hundred forty-one cases in the Sydney West Advanced Pelvic Surgery Unit. J Minim Invasive Gynecol. 2010;17(2):186-91.

79. Wright JD, Devine P, Shah M, Gaddipati S, Lewin SN, Simpson LL, et al. Morbidity and mortality of peripartum hysterectomy. Obstet Gynecol. 2010;115(6):1187-93.
80. Anpalagan A, Hardas G, Merkur H. Inadvertent cystotomy at laparoscopic hysterectomy - Sydney West Advanced Pelvic Surgery (SWAPS) Unit January 2001 to June 2009. Australian and New Zealand Journal of Obstetrics and Gynaecology. 2011;51(4):325-7.
81. Brummer THI, Jalkanen J, Fraser J, Heikkinen AM, Kauko M, J MA, et al. FINHYST, a prospective study of 5279 hysterectomies: Complications and their risk factors. Human Reproduction. 2011;26(7):1741-51.
82. Doganay M, Yildiz Y, Tonguc E, Var T, Karayalcin R, Eryilmaz OG, et al. Abdominal, vaginal and total laparoscopic hysterectomy: perioperative morbidity. Arch Gynecol Obstet. 2011;284(2):385-9.
83. Jung MH, Lee BY. Transumbilical single-port laparoscopic-assisted vaginal hysterectomy via 12-mm trocar incision site. J Laparoendosc Adv Surg Tech A. 2011;21(7):599-602.
84. Kavallaris A, Kalogiannidis I, Chalvatzas N, Hornemann A, Beyer D, Georgiev I, et al. Laparoscopic-assisted vaginal hysterectomy with and without laparoscopic transsection of the uterine artery: An analysis of 1,255 cases. Archives of Gynecology and Obstetrics. 2011;284(2):379-84.
85. Lee YS, Lee JH, Choi JS, Son CE, Jeon SW, Kim JT, et al. Accessory polar renal artery encountered in transperitoneal systemic laparoscopic paraaortic lymphadenectomy. Eur J Gynaecol Oncol. 2011;32(1):87-90.
86. Ozdemir E, Ozturk U, Celen S, Sucak A, Gunel M, Guney G, et al. Urinary complications of gynecologic surgery: iatrogenic urinary tract system injuries in obstetrics and gynecology operations. Clin Exp Obstet Gynecol. 2011;38(3):217-20.
87. Song T, Kim TJ, Kang H, Lee YY, Choi CH, Lee JW, et al. A review of the technique and complications from 2,012 cases of Laparoscopically Assisted Vaginal Hysterectomy at a single institution. Australian and New Zealand Journal of Obstetrics and Gynaecology. 2011;51(3):239-43.
88. Yada-Hashimoto N, Onoue M, Yoshimi K, Hisa T, Kodama M, Otsuka H, et al. Total laparoscopic hysterectomy in patients with previous abdominal surgery. Arch Gynecol Obstet. 2011;284(6):1467-71.
89. Acharya S, Uprety DK, Pokharel HP, Amatya R, Rai R. Cesarean section without urethral catheterization: A randomized control trial. Kathmandu University Medical Journal. 2012;10(38):18-22.
90. Al-Shahrani M. Bladder injury during Cesarean section: A case control study for 10 years. Bahrain Medical Bulletin. 2012;34(3).
91. Cho HY, Kang SW, Kim HB, Park SH, Park ST. Prophylactic adnexectomy along with vaginal hysterectomy for benign pathology. Arch Gynecol Obstet. 2012;286(5):1221-5.
92. Grosse-Drieling D, Schlutius JC, Altgassen C, Kelling K, Theben J. Laparoscopic supracervical hysterectomy (LASH), a retrospective study of 1,584 cases regarding intra- and perioperative complications. Archives of Gynecology and Obstetrics. 2012;285(5):1391-6.
93. Khan B, Khan B, Sultana R, Bashir R, Deeba F. A ten year review of emergency peripartum hysterectomy in a tertiary care hospital. J Ayub Med Coll Abbottabad. 2012;24(1):14-7.
94. Kobayashi E, Nagase T, Fujiwara K, Hada T, Ota Y, Takaki Y, et al. Total laparoscopic hysterectomy in 1253 patients using an early ureteral identification technique. Journal of Obstetrics and Gynaecology Research. 2012;38(9):1194-200.
95. Lee JS, Choe JH, Lee HS, Seo JT. Urologic complications following obstetric and gynecologic surgery. Korean J Urol. 2012;53(11):795-9.
96. Mueller A, Boosz A, Koch M, Jud S, Faschingbauer F, Schrauder M, et al. The Hohl instrument for optimizing total laparoscopic hysterectomy: Results of more than 500 procedures in a university training center. Archives of Gynecology and Obstetrics. 2012;285(1):123-7.
97. Rao D, Yu H, Zhu H, Duan P. The diagnosis and treatment of iatrogenic ureteral and bladder injury caused by traditional gynaecology and obstetrics operation. Arch Gynecol Obstet. 2012;285(3):763-5.
98. Sandberg EM, Cohen SL, Hurwitz S, Einarsson JI. Utility of cystoscopy during hysterectomy. Obstet Gynecol. 2012;120(6):1363-70.
99. Teerapong S, Rungaramsin P, Tanprasertkul C, Bhamarapravatana K, Suwannarurk K. Major complication of gynaecological laparoscopy in Police General Hospital: a 4-year experience. J Med Assoc Thai. 2012;95(11):1378-83.
100. Tuuli MG, Odibo AO, Fogertey P, Roehl K, Stamilio D, MacOnes GA. Utility of the bladder flap at cesarean delivery: A randomized controlled trial. Obstetrics and Gynecology. 2012;119(4):815-21.
101. Choi YS, Park JN, Oh YS, Sin KS, Choi J, Eun DS. Single-port vs. conventional multi-port access laparoscopy-assisted vaginal hysterectomy: comparison of surgical outcomes and complications. Eur J Obstet Gynecol Reprod Biol. 2013;169(2):366-9.
102. Gasim T, Al Jama FE, Rahman MS, Rahman J. Multiple repeat cesarean sections: operative difficulties, maternal complications and outcome. J Reprod Med. 2013;58(7-8):312-8.
103. Jo EJ, Kim TJ, Lee YY, Choi CH, Lee JW, Bae DS, et al. Laparoendoscopic single-site surgery with hysterectomy in patients with prior cesarean section: comparison of surgical outcomes with bladder dissection techniques. J Minim Invasive Gynecol. 2013;20(2):160-5.
104. Litta P, Saccardi C, Conte L, Florio P. Reverse hysterectomy: another technique for performing a laparoscopic hysterectomy. J Minim Invasive Gynecol. 2013;20(5):631-6.
105. Makinen J, Brummer T, Jalkanen J, Heikkinen AM, Fraser J, Tomas E, et al. Ten years of progress--improved hysterectomy outcomes in Finland 1996-2006: a longitudinal observation study. BMJ Open. 2013;3(10):e003169.
106. Merritt AJ, Crosbie EJ, Charova J, Achiampong J, Zommere I, Winter-Roach B, et al. Prophylactic pre-operative bilateral ureteric catheters for major gynaecological surgery. Arch Gynecol Obstet. 2013;288(5):1061-6.
107. Pundir J, Krishnan N, Siozos A, Uwins C, Kopeika J, Khalaf Y, et al. Peri-operative morbidity associated with abdominal myomectomy for very large fibroid uteri. European Journal of Obstetrics and Gynecology and Reproductive Biology. 2013;167(2):219-24.
108. Sheth SS. Vaginal hysterectomy in women with a history of 2 or more cesarean deliveries. Int J Gynaecol Obstet. 2013;122(1):70-4.
109. Dutta DK, Dutta I. Abdominal hysterectomy: a new approach for conventional procedure. J Clin Diagn Res. 2014;8(4):OC15-8.
110. Gowri V, Gonsalvez H, Al Riyami N, Machado L, Al Dugghaishi T, Mathew M. Peer review and audit of morbidity after three or more caesarean sections. Journal of Taibah University Medical Sciences. 2014;9(3):194-7.
111. Han L, Cao R, Jiang JY, Xi Y, Li XC, Yu GH. Preset ureter catheter in laparoscopic radical hysterectomy of cervical cancer. Genet Mol Res. 2014;13(2):3638-45.
112. Lanowska M, Brink-Spalink V, Mangler M, Grittner U, von Tucher E, Schneider A, et al. Vaginal-assisted laparoscopic radical hysterectomy (VALRH) versus laparoscopic-assisted radical vaginal hysterectomy (LARVH) in the treatment of cervical cancer: surgical results and oncologic outcome. Arch Gynecol Obstet. 2014;289(6):1293-300.
113. Mamik MM, Antosh D, White DE, Myers EM, Abernethy M, Rahimi S, et al. Risk factors for lower urinary tract injury at the time of hysterectomy for benign reasons. Int Urogynecol J. 2014;25(8):1031-6.
114. Manusook S, Suwannarurk K, Pongrojpaw D, Bhamarapravatana K. Maylard incision in gynecologic surgery: 4-year experience in Thammasat University Hospital. J Med Assoc Thai. 2014;97 Suppl 8(Supplement 8):S102-7.
115. Nguyen ML, Stevens E, LaFargue CJ, Karsy M, Pua TL, Gorelick C, et al. Routine cystoscopy after robotic gynecologic oncology surgery. JSLS. 2014;18(3):Jul-Sep.
116. Park JY, Nam JH. Laparotomy conversion rate of laparoscopic radical hysterectomy for early-stage cervical cancer in a consecutive series without case selection. Annals of Surgical Oncology. 2014;21(9):3030-5.
117. Rizzuto I, Odejinmi F, Al-Samarrai M. Can body mass index influence the outcome of a laparoscopic hysterectomy? Journal of Gynecologic Surgery. 2014;30(2):74-80.
118. Zia S, Rafique M. Intra-operative complications increase with successive number of cesarean sections: Myth or fact? Obstet Gynecol Sci. 2014;57(3):187-92.
119. Bogani G, Cromi A, Serati M, Di Naro E, Casarin J, Marconi N, et al. Hysterectomy in patients with previous cesarean section: Comparison between laparoscopic and vaginal approaches. European Journal of Obstetrics Gynecology and Reproductive Biology. 2015;184:53-7.
120. Garabedian C, Merlot B, Bresson L, Tresch E, Narducci F, Leblanc E. Minimally invasive surgical management of early-stage cervical cancer: an analysis of the risk factors of surgical complications and of oncologic outcomes. Int J Gynecol Cancer. 2015;25(4):714-21.
121. Kaplanoglu M, Bulbul M, Kaplanoglu D, Bakacak SM. Effect of multiple repeat cesarean sections on maternal morbidity: data from southeast Turkey. Med Sci Monit. 2015;21:1447-53.
122. Odejinmi F, Maclaran K, Agarwal N. Laparoscopic treatment of uterine fibroids: a comparison of peri-operative outcomes in laparoscopic hysterectomy and myomectomy. Arch Gynecol Obstet. 2015;291(3):579-84.
123. Tan-Kim J, Menefee SA, Reinsch CS, O'Day CH, Bebchuk J, Kennedy JS, et al. Laparoscopic Hysterectomy and Urinary Tract Injury: Experience in a Health Maintenance Organization. J Minim Invasive Gynecol. 2015;22(7):1278-86.
124. Yim GW, Kim SW, Nam EJ, Kim S, Kim YT. Perioperative complications of robot-assisted laparoscopic surgery using three robotic arms at a single institution. Yonsei Medical Journal. 2015;56(2):474-81.
125. Dolanbay M, Kutuk MS, Ozgun MT, Uludag S, Sahin Y. Laparoscopically-assisted vaginal hysterectomy for enlarged uterus: operative outcomes and the learning curve. Ginekol Pol. 2016;87(5):333-7.
126. Kang HW, Lee JW, Kim HY, Kim BW, Moon CS. Total laparoscopic hysterectomy via suture and ligation technique. Obstet Gynecol Sci. 2016;59(1):39-44.
127. Liu S, Shi R, Xie Y, Sun H. A novel laparoscopic surgical technique for severe adenomyoma. Clin Exp Obstet Gynecol. 2016;43(5):656-60.
128. Maclaran K, Agarwal N, Odejinmi F. Perioperative outcomes in laparoscopic hysterectomy: identifying surgical risk factors. Gynecological Surgery. 2016;13(2):75-82.
129. Moores KL, Bentick B. Gynaecological laparoscopic injuries: a 10-year retrospective review at a District General Hospital NHS Trust. Gynecological Surgery. 2016;13(2):125-30.
130. Tinelli R, Cicinelli E, Tinelli A, Bettocchi S, Angioni S, Litta P. Laparoscopic treatment of early-stage endometrial cancer with and without uterine manipulator: Our experience and review of literature. Surgical Oncology. 2016;25(2):98-103.
131. Chen CH, Chen HH, Liu WM. Complication reports for robotic surgery using three arms by a single surgeon at a single institution. Journal of Minimal Access Surgery. 2017;13(1):22-8.
132. Clave H, Clave A. Safety and Efficacy of Advanced Bipolar Vessel Sealing in Vaginal Hysterectomy: 1000 Cases. J Minim Invasive Gynecol. 2017;24(2):272-9.
133. Jeung IC, Lee YS, Song MJ, Park EK. Laparoendoscopic Single-Site Total Laparoscopic Hysterectomy: Clinical Factors that Affect Operative Times and Techniques to Overcome Difficulties. Journal of Minimally Invasive Gynecology. 2017;24(4):617-25.
134. Lim S, Lee S, Choi J, Chon S, Lee K, Shin J. Safety of total laparoscopic hysterectomy in patients with prior cesarean section. J Obstet Gynaecol Res. 2017;43(1):196-201.
135. Mahmood S, Iqbal N, Aslam S. Assessment of urological complications following obstetric and gynaecological surgery - A five years review. Pakistan Journal of Medical and Health Sciences. 2017;11(3):1068-71.
136. Satitniramai S, Manonai J. Urologic injuries during gynecologic surgery, a 10-year review. J Obstet Gynaecol Res. 2017;43(3):557-63.
137. Singla A, Mundhra R, Phogat L, Mehta S, Rajaram S. Emergency Peripartum Hysterectomy: Indications and Outcome in a Tertiary Care Setting. J Clin Diagn Res. 2017;11(3):QC01-QC3.
138. Uyanikoglu H, Karahan MA, Turp AB, Agar M, Tasduzen ME, Sak S, et al. Are multiple repeated cesarean sections really as safe? Journal of Maternal-Fetal and Neonatal Medicine. 2017;30(4):482-5.
139. Yaman Tunc S, Agacayak E, Sak S, Basaranoglu S, Goruk NY, Turgut A, et al. Multiple repeat caesarean deliveries: do they increase maternal and neonatal morbidity? Journal of Maternal-Fetal and Neonatal Medicine. 2017;30(6):739-44.
140. Benson CR, Thompson S, Li G, Asafu-Adjei D, Brandes SB. Bladder and ureteral injuries during benign hysterectomy: an observational cohort analysis in New York State. World J Urol. 2018;07:07.
141. Blackwell RH, Kirshenbaum EJ, Shah AS, Kuo PC, Gupta GN, Turk TMT. Complications of Recognized and Unrecognized Iatrogenic Ureteral Injury at Time of Hysterectomy: A Population Based Analysis. Journal of Urology. 2018;199(6):1540-5.
142. Jain N, Kamra J, Chabbra A. Rising trend of laparoscopic hysterectomy over abdominal hysterectomy: A comparative study. World Journal of Laparoscopic Surgery. 2018;11(2):59-63.
143. Koroglu N, Cetin BA, Turan G, Yildirim GY, Akca A, Gedikbasi A. Characteristics of total laparoscopic hysterectomy among women with or without previous cesarean section: Retrospective analysis. Sao Paulo Medical Journal. 2018;136(5):385-9.
144. Li F, Guo H, Qiu H, Liu S, Wang K, Yang C, et al. Urological complications after radical hysterectomy with postoperative radiotherapy and radiotherapy alone for cervical cancer. Medicine (Baltimore). 2018;97(13):e0173.
145. Petersen SS, Doe S, Rubinfeld I, Davydova Y, Buekers T, Sangha R. Rate of Urologic Injury with Robotic Hysterectomy. J Minim Invasive Gynecol. 2018;25(5):867-71.
146. Alanwar A, Al-Sayed HM, Ibrahim AM, Elkotb AM, Abdelshafy A, Abdelhadi R, et al. Urinary tract injuries during cesarean section in patients with morbid placental adherence: retrospective cohort study. J Matern Fetal Neonatal Med. 2019;32(9):1461-7.
147. Inan AH, Budak A, Beyan E, Kanmaz AG. The incidence, causes, and management of lower urinary tract injury during total laparoscopic hysterectomy. J Gynecol Obstet Hum Reprod. 2019;48(1):45-9.
148. Melnikoff AK, Doo DW, Cohen AC, Landers E, Walters-Haygood C, McGwin G, et al. Timing of robotic hysterectomy after cervical excisional procedure. Int J Gynecol Cancer. 2019;29(7):1110-5.
149. Otkjaer AM, Jorgensen HL, Clausen TD, Krebs L. Maternal short-term complications after planned cesarean delivery without medical indication: A registry-based study. Acta Obstetricia et Gynecologica Scandinavica. 2019;98(7):905-12.
150. Sinha R, Swarnasree G, Rupa B, Madhumathi S. Laparoscopic hysterectomy for large uteri: Outcomes and techniques. J Minim Access Surg. 2019;15(1):8-13.
151. Sirota I, Tomita SA, Dabney L, Weinberg A, Chuang L. Overcoming barriers to vaginal hysterectomy: An analysis of perioperative outcomes. J Turk Ger Gynecol Assoc. 2019;20(1):8-14.
152. Songdegeroth KE, Wan L, Rampersad RM, Stout MJ, Macones GA, Cahill AG, et al. Risk of Maternal Morbidity with Increasing Number of Cesareans. Am J Perinatol. 2019;36(4):346-51.
153. Tayeh NK, Kareem NK, Fawzi HA. Bladder injury as a complication of cesarean deliveries and peripartum hysterectomy. Indian Journal of Public Health Research and Development. 2019;10(4):467-71.

Appendix 5. Meta-analyses by procedure type and organ injured.

Bladder injury	Ureteric injury

Caesarean section

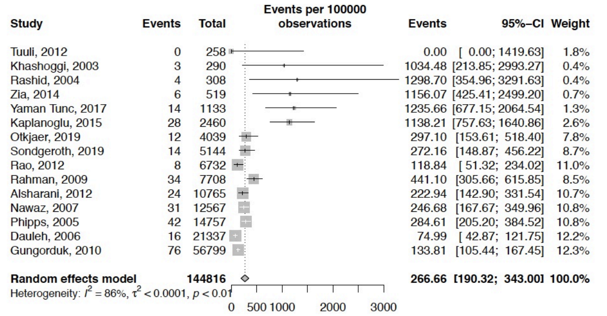

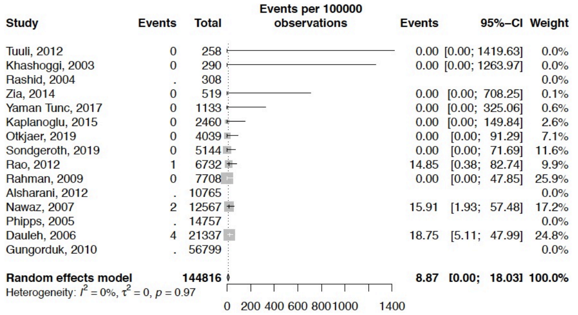

Hysterectomy, open abdominal (benign)

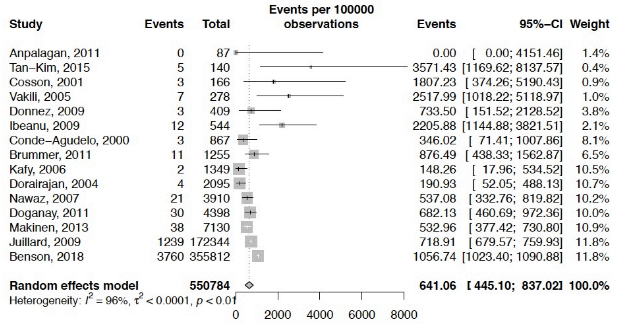

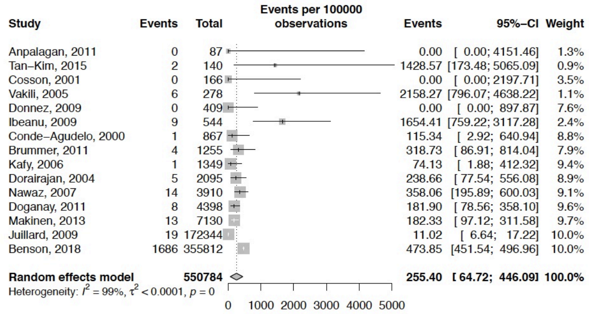

Hysterectomy, open abdominal (any histology)

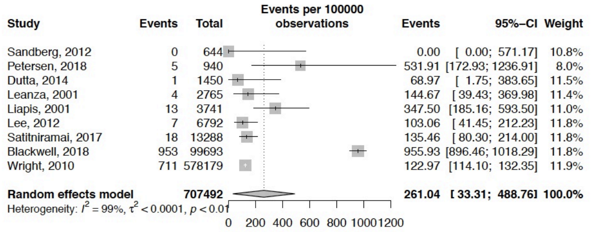

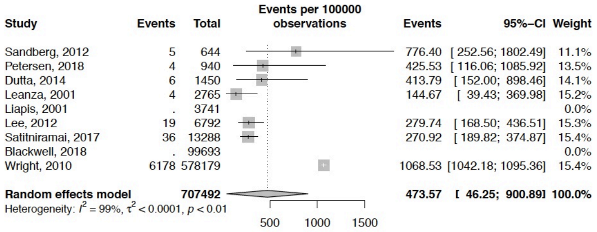

Hysterectomy, open abdominal (malignant)

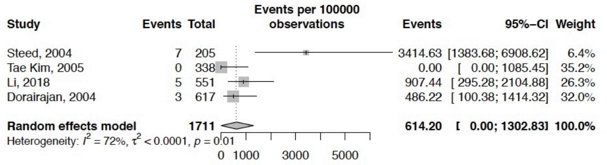

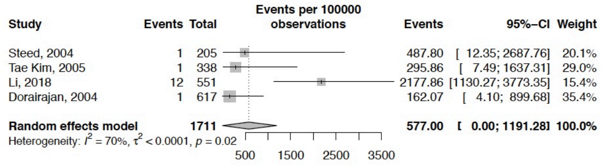

Hysterectomy, open abdominal (peripartum)

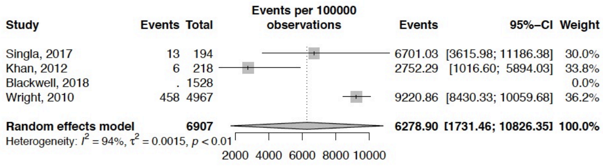

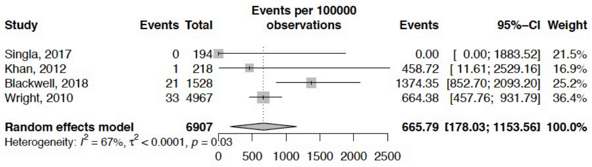

Hysterectomy, vaginal (benign)

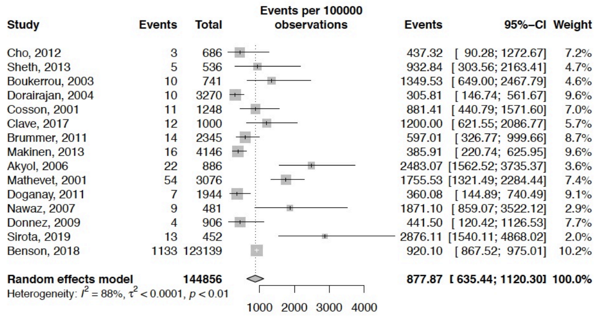

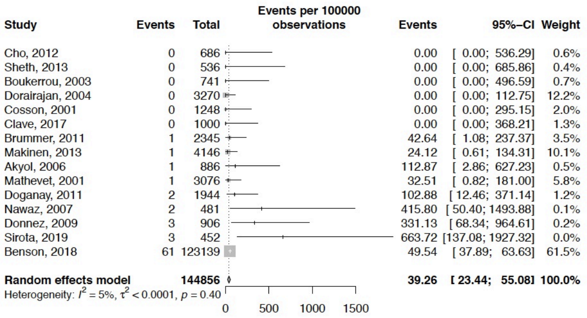

Hysterectomy, vaginal (any histology)

Study	Events	Total	Events per 100000 observations	95%-CI	Weight
Sandberg, 2012	2	250	800.00	[97.03; 2859.83]	1.6%
Liapis, 2001	.	1381			0.0%
Petersen, 2018	0	304	0.00	[0.00; 1206.11]	9.5%
Leanza, 2001	2	373	536.19	[65.00; 1923.40]	3.5%
Lee, 2012	16	5182	308.76	[176.58; 500.93]	85.4%
Blackwell, 2018	.	45002			0.0%
**Random effects model**		**52492**	**295.49**	**[155.90; 435.09]**	**100.0%**

Heterogeneity: $I^2 = 0\%$, $\tau^2 = 0$, $p = 0.41$

0 500 1000 1500 2000 2500

Study	Events	Total	Events per 100000 observations	95%-CI	Weight
Sandberg, 2012	0	250	0.00	[0.00; 1464.72]	4.0%
Liapis, 2001	0	1381	0.00	[0.00; 266.76]	27.2%
Petersen, 2018	1	304	328.95	[8.33; 1819.06]	3.0%
Leanza, 2001	1	373	268.10	[6.79; 1484.62]	4.3%
Lee, 2012	5	5182	96.49	[31.34; 225.03]	28.9%
Blackwell, 2018	100	45002	222.21	[180.84; 270.20]	32.5%
**Random effects model**		**52492**	**121.70**	**[4.88; 238.53]**	**100.0%**

Heterogeneity: $I^2 = 75\%$, $\tau^2 < 0.0001$, $p < 0.01$

0 500 1000 1500

Hysterectomy, lap. (benign)

Study	Events	Total	Events per 100000 observations	95%-CI	Weight
Karaman, 2007	0	552	0.00	[0.00; 666.05]	7.5%
Anpalagan, 2011	14	991	1412.71	[774.44; 2358.94]	6.8%
Koroglu, 2018	2	504	396.83	[48.09; 1426.06]	7.2%
Bojahr, 2006	3	1706	175.85	[36.28; 513.04]	7.6%
Mueller, 2012	4	567	705.47	[192.54; 1796.36]	6.9%
Grosse–Drieling, 2012	4	1584	252.53	[68.85; 645.29]	7.5%
Wang, 2010	12	574	2090.59	[1084.79; 3623.35]	5.9%
Chopin, 2009	14	1460	958.90	[525.21; 1603.65]	7.3%
Inan, 2019	7	547	1279.71	[516.01; 2618.83]	6.4%
Leonard, 2007	.	1300			0.0%
Lafay Pillet, 2009	15	1501	999.33	[560.37; 1642.90]	7.2%
Brummer, 2011	17	1679	1012.51	[590.90; 1616.19]	7.3%
Wattiez, 2002	19	1647	1153.61	[695.94; 1795.67]	7.2%
Makinen, 2013	47	4113	1142.72	[840.80; 1516.71]	7.5%
Benson, 2018	830	31389	2644.24	[2469.54; 2827.77]	7.6%
**Random effects model**		**50114**	**997.13**	**[400.51; 1593.74]**	**100.0%**

Heterogeneity: $I^2 = 97\%$, $\tau^2 = 0.0001$, $p < 0.01$

0 5001000 2000 3000

Study	Events	Total	Events per 100000 observations	95%-CI	Weight
Karaman, 2007	0	552	0.00	[0.00; 666.05]	7.0%
Anpalagan, 2011	0	991	0.00	[0.00; 371.55]	8.4%
Koroglu, 2018	1	504	198.41	[5.02; 1100.48]	5.3%
Bojahr, 2006	1	1706	58.62	[1.48; 326.15]	8.7%
Mueller, 2012	1	567	176.37	[4.47; 978.70]	5.8%
Grosse–Drieling, 2012	1	1584	63.13	[1.60; 351.24]	8.6%
Wang, 2010	2	574	348.43	[42.22; 1252.94]	4.3%
Chopin, 2009	3	1460	205.48	[42.39; 599.31]	7.3%
Inan, 2019	4	547	731.26	[199.59; 1861.66]	2.6%
Leonard, 2007	4	1300	307.69	[83.90; 785.93]	6.4%
Lafay Pillet, 2009	5	1501	333.11	[108.25; 775.65]	6.5%
Brummer, 2011	5	1679	297.80	[96.76; 693.58]	6.9%
Wattiez, 2002	6	1647	364.30	[133.80; 791.23]	6.5%
Makinen, 2013	31	4113	753.71	[512.67; 1068.14]	6.9%
Benson, 2018	158	31389	503.36	[428.09; 588.00]	8.9%
**Random effects model**		**50114**	**262.38**	**[125.84; 398.92]**	**100.0%**

Heterogeneity: $I^2 = 85\%$, $\tau^2 < 0.0001$, $p < 0.01$

0 500 1000 1500

Hysterectomy, lap. (any histology)

Study	Events	Total	Events per 100000 observations	95%-CI	Weight
Lee, 2012	0	1625	0.00	[0.00; 226.75]	16.4%
Mahdavi, 2006	1	159	628.93	[15.92; 3454.21]	2.3%
Lim, 2017	1	482	207.47	[5.25; 1150.48]	10.1%
Sharon, 2006	1	480	208.33	[5.27; 1155.25]	10.0%
Ng, 2007	2	503	397.61	[48.19; 1428.88]	7.5%
Kang, 2016	6	746	804.29	[295.72; 1742.33]	6.3%
Petersen, 2018	3	782	383.63	[79.18; 1117.01]	9.5%
Kobayashi, 2012	6	1253	478.85	[175.93; 1039.32]	10.6%
Sandberg, 2012	7	1011	692.38	[278.81; 1421.34]	8.1%
Satitniramai, 2017	4	2131	187.71	[51.17; 479.90]	14.9%
Seman, 2001	.	436			0.0%
O'Hanlan, 2007	13	830	1566.27	[836.54; 2663.47]	4.3%
Blackwell, 2018	.	16584			0.0%
**Random effects model**		**27022**	**375.14**	**[172.88; 577.39]**	**100.0%**

Heterogeneity: $I^2 = 72\%$, $\tau^2 < 0.0001$, $p < 0.01$

0 5001000 2000 3000

Study	Events	Total	Events per 100000 observations	95%-CI	Weight
Lee, 2012	0	1625	0.00	[0.00; 226.75]	9.5%
Mahdavi, 2006	0	159	0.00	[0.00; 2293.34]	5.2%
Lim, 2017	0	482	0.00	[0.00; 762.41]	8.8%
Sharon, 2006	1	480	208.33	[5.27; 1155.25]	8.1%
Ng, 2007	1	503	198.81	[5.03; 1102.66]	8.2%
Kang, 2016	3	746	402.14	[83.01; 1170.70]	7.8%
Petersen, 2018	4	782	511.51	[139.54; 1304.45]	7.5%
Kobayashi, 2012	4	1253	319.23	[87.05; 815.33]	8.6%
Sandberg, 2012	4	1011	395.65	[107.90; 1009.89]	8.2%
Satitniramai, 2017	6	2131	281.56	[103.39; 611.82]	9.1%
Seman, 2001	6	436	1376.15	[506.65; 2971.10]	4.1%
O'Hanlan, 2007	10	830	1204.82	[579.23; 2204.51]	5.9%
Blackwell, 2018	182	16584	1097.44	[944.49; 1267.89]	9.3%
**Random effects model**		**27022**	**416.78**	**[126.57; 706.99]**	**100.0%**

Heterogeneity: $I^2 = 92\%$, $\tau^2 < 0.0001$, $p < 0.01$

0 500 1000 1500 2000 2500

Hysterectomy, lap. (malignant)

Study	Events	Total	Events per 100000 observations	95%-CI	Weight
Ghezzi, 2006	1	101	990.10	[25.06; 5393.23]	12.0%
Park, 2014	3	260	1153.85	[238.59; 3334.81]	16.6%
Chen, 2008	5	290	1724.14	[562.13; 3977.48]	15.0%
Yan, 2009	6	117	5128.21	[1904.95; 10828.01]	4.5%
Garabedian, 2015	3	170	1764.71	[365.41; 5070.38]	11.7%
Tinelli, 2016	3	110	2727.27	[566.00; 7763.68]	6.9%
Xu, 2007	9	317	2839.12	[1306.27; 5320.93]	12.7%
Han, 2014	0	176	0.00	[0.00; 2074.14]	20.7%
**Random effects model**		**1541**	**1553.43**	**[610.44; 2496.43]**	**100.0%**

Heterogeneity: $I^2 = 59\%$, $\tau^2 < 0.0001$, $p = 0.02$

0 2000 4000 6000 8000 10000

Study	Events	Total	Events per 100000 observations	95%-CI	Weight
Ghezzi, 2006	0	101	0.00	[0.00; 3586.46]	12.7%
Park, 2014	1	260	384.62	[9.74; 2124.19]	23.4%
Chen, 2008	1	290	344.83	[8.73; 1906.18]	25.2%
Yan, 2009	1	117	854.70	[21.64; 4670.09]	9.4%
Garabedian, 2015	2	170	1176.47	[142.79; 4185.01]	9.8%
Tinelli, 2016	4	110	3636.36	[999.52; 9049.20]	2.7%
Xu, 2007	5	317	1577.29	[514.07; 3642.28]	12.5%
Han, 2014	6	176	3409.09	[1261.16; 7272.17]	4.3%
**Random effects model**		**1541**	**813.94**	**[221.81; 1406.08]**	**100.0%**

Heterogeneity: $I^2 = 38\%$, $\tau^2 < 0.0001$, $p = 0.13$

0 2000 4000 6000 8000

Hysterectomy, lap. assisted vaginal (benign)

Study	Events	Total	Events per 100000 observations	95%-CI	Weight
Jung, 2011	0	458	0.00	[0.00; 802.20]	16.5%
Karaman, 2007	0	542	0.00	[0.00; 678.29]	17.6%
Ark, 2009	1	367	272.48	[6.90; 1508.74]	11.0%
Ibeanu, 2009	2	61	3278.69	[399.56; 11347.22]	0.3%
Johnston, 2007	2	69	2898.55	[352.98; 10081.54]	0.4%
Dolanbay, 2016	2	184	1086.96	[131.91; 3871.11]	2.5%
Kafy, 2006	2	223	896.86	[108.80; 3202.04]	3.5%
Chang, 2005	2	225	888.89	[107.83; 3173.91]	3.6%
Milad, 2001	3	105	2857.14	[593.13; 8123.19]	0.6%
Choi, 2013	3	250	1200.00	[248.16; 3466.66]	3.0%
Lee, 2009	3	512	585.94	[121.00; 1702.74]	8.8%
Teerapong, 2012	4	101	3960.40	[1089.45; 9830.50]	0.4%
Kavallaris, 2011	11	1255	876.49	[438.33; 1562.87]	11.4%
Soong, 2007	30	7725	388.35	[262.17; 553.93]	20.2%
**Random effects model**		**12077**	**444.74**	**[190.31; 699.17]**	**100.0%**

Heterogeneity: $I^2 = 57\%$, $\tau^2 < 0.0001$, $p < 0.01$

0 2000 4000 6000 8000

Study	Events	Total	Events per 100000 observations	95%-CI	Weight
Jung, 2011	0	458	0.00	[0.00; 802.20]	4.0%
Karaman, 2007	0	542	0.00	[0.00; 678.29]	5.6%
Ark, 2009	0	367	0.00	[0.00; 1000.11]	2.6%
Ibeanu, 2009	0	61	0.00	[0.00; 5868.12]	0.1%
Johnston, 2007	0	69	0.00	[0.00; 5205.81]	0.1%
Dolanbay, 2016	0	184	0.00	[0.00; 1984.86]	0.7%
Kafy, 2006	0	223	0.00	[0.00; 1640.60]	1.0%
Chang, 2005	0	225	0.00	[0.00; 1626.14]	1.0%
Milad, 2001	0	105	0.00	[0.00; 3452.22]	0.2%
Choi, 2013	0	250	0.00	[0.00; 1464.72]	1.2%
Lee, 2009	0	512	0.00	[0.00; 717.90]	5.0%
Teerapong, 2012	3	101	2970.30	[616.79; 8435.69]	0.0%
Kavallaris, 2011	2	1255	159.36	[19.31; 574.48]	7.5%
Soong, 2007	8	7725	103.56	[44.72; 203.95]	71.1%
**Random effects model**		**12077**	**86.55**	**[26.08; 147.01]**	**100.0%**

Heterogeneity: $I^2 = 0\%$, $\tau^2 = 0$, $p = 0.97$

0 2000 4000 6000 8000

Hysterectomy, lap. assisted vaginal (any histology)

Study	Events	Total	Events per 100000 observations	95%-CI	Weight
Roman, 2006	0	418	0.00	[0.00; 878.62]	14.4%
Koh, 2004	5	2006	249.25	[80.98; 580.71]	16.6%
Parkar, 2004	6	149	4026.85	[1491.89; 8558.48]	0.6%
Lee, 2012	8	2891	276.72	[119.54; 544.52]	17.0%
Tian, 2007	10	2174	459.98	[220.79; 844.29]	15.3%
Shen, 2003	11	2702	407.11	[203.40; 727.25]	16.2%
Kyung, 2008	15	1178	1273.34	[714.39; 2091.50]	8.7%
Song, 2011	26	2012	1292.25	[845.83; 1887.74]	11.1%
Blackwell, 2018	.	27158			0.0%
**Random effects model**		**40688**	**505.75**	**[247.77; 763.73]**	**100.0%**

Heterogeneity: $I^2 = 79\%$, $\tau^2 < 0.0001$, $p < 0.01$

0 2000 4000 6000 8000

Study	Events	Total	Events per 100000 observations	95%-CI	Weight
Roman, 2006	0	418	0.00	[0.00; 878.62]	10.1%
Koh, 2004	0	2006	0.00	[0.00; 183.72]	12.6%
Parkar, 2004	0	149	0.00	[0.00; 2445.36]	4.1%
Lee, 2012	7	2891	242.13	[97.40; 498.24]	11.8%
Tian, 2007	2	2174	92.00	[11.14; 331.92]	12.3%
Shen, 2003	4	2702	148.04	[40.35; 378.60]	12.2%
Kyung, 2008	1	1178	84.89	[2.15; 472.06]	12.0%
Song, 2011	1	2012	49.70	[1.26; 276.61]	12.5%
Blackwell, 2018	245	27158	902.13	[793.11; 1021.83]	12.4%
**Random effects model**		**40688**	**186.17**	**[0.00; 414.63]**	**100.0%**

Heterogeneity: $I^2 = 96\%$, $\tau^2 < 0.0001$, $p < 0.01$

0 500 1000 1500 2000

Hysterectomy, robot assisted (any histology)

Study	Events	Total	Events per 100000 observations	95%-CI	Weight
Sandberg, 2012	0	77	0.00	[0.0; 4677.81]	2.4%
Nguyen, 2014	0	229	0.00	[0.0; 1597.96]	20.6%
Petersen, 2018	3	1088	275.74	[56.9; 803.68]	77.0%
**Random effects model**		**1394**	**212.28**	**[0.0; 485.67]**	**100.0%**

Heterogeneity: $I^2 = 0\%$, $\tau^2 = 0$, $p = 0.71$

0 1000 2000 3000 4000

Study	Events	Total	Events per 100000 observations	95%-CI	Weight
Sandberg, 2012	1	77	1298.70	[32.87; 7024.71]	4.4%
Nguyen, 2014	0	229	0.00	[0.00; 1597.96]	42.5%
Petersen, 2018	7	1088	643.38	[259.05; 1321.10]	53.1%
**Random effects model**		**1394**	**398.16**	**[0.00; 939.11]**	**100.0%**

Heterogeneity: $I^2 = 37\%$, $\tau^2 < 0.0001$, $p = 0.20$

0 1000 3000 5000 7000

Appendix 6. Risk factors for urological injury

Year	First author	Surgeon inexperience or low volume (n=18)	Prior LSCS (n=17)	Prior pelvic surgery (n=14)	Adhesions (n=10)	Large uterus † (n=10)	Endome -triosis (n=8)	Cancer (n=5)	Radiation (n=5)	Haemo -rrhage (n=5)	BMI low or high (n=5)	Placental adhesion disorder (n=4)	Cocomitant surgery (n=4)	Emergency LSCS (n=2)
2000	Conde-Agudelo (12)												1	
2001	Cosson (13)													
2001	Leanza (14)													
2001	Liapis (15)			1				1	1		1			
2001	Mathevet (16)			1				1	1					
2001	Milad (17)													
2001	Seman (18)													
2002	Wattiez (19)					1	1			1				
2003	Boukerrou (20)		1											
2003	Khashoggi (21)													
2003	Shen (22)	1			1									
2004	Dorairajan (23)													
2004	Koh (24)													
2004	Parkar (25)													
2004	Rashid (26)		1								1			
2004	Steed (27)	1												
2005	Chang (28)													
2005	Phipps (29)		1	1							1			1
2005	Tae Kim (30)													
2005	Vakili (31)												1	
2006	Akyol (32)		1		1	1								
2006	Bojahr (33)	1		1		1								
2006	Dauleh (34)													
2006	Ghezzi (35)													
2006	Kafy (36)													
2006	Mahdavi (37)													
2006	Roman (38)													
2006	Sharon (39)	1												
2007	Johnston (40)	1		1			1				1			
2007	Karaman (41)													
2007	Leonard (42)	1												
2007	Nawaz (43)		1									1		
2007	Ng (44)													
2007	O'Hanlan (45)													
2007	Soong (46)			1	1	1	1			1				
2007	Tian (47)													
2007	Xu (48)	1											1	
2008	Chen (49)	1												
2008	Kyung (50)		1											
2009	Ark (51)													
2009	Chopin (52)													
2009	Donnez (53)													
2009	Ibeanu (54)					1				1			1	
2009	Juillard (55)													
2009	Lafay Pillet (56)	1	1	1	1									
2009	Lee (57)													
2009	Rahman (58)	1	1	1	1									
2009	Yan (59)													
2010	Gungorduk (60)		1	1	1									
2010	Wang (61)	1												
2010	Wright (62)											1		
2011	Anpalagan (63)		1			1	1							
2011	Brummer (64)				1	1	1							
2011	Doganay (65)													
2011	Jung (66)													
2011	Kavallaris (67)									1				
2011	Song (68)													
2012	Alsharani (69)		1									1		1
2012	Cho (70)													
2012	Grosse-Drieling (71)			1										
2012	Khan (72)													
2012	Kobayashi (73)			1										
2012	Lee (74)													
2012	Mueller (75)													
2012	Rao (76)	1			1	1		1	1					
2012	Sandberg (77)	1			1									
2012	Teerapong (78)													
2012	Tuuli (79)													
2013	Choi (80)													
2013	Makinen (81)	1												
2013	Sheth (82)													
2014	Dutta (83)									1				
2014	Han (84)													
2014	Nguyen (85)													
2014	Park (86)	1												
2014	Zia (87)		1											
2015	Garabedian (88)					1			1					
2015	Kaplanoglu (89)		1											
2015	Tan-Kim (90)						1				1			
2016	Dolanbay (91)													
2016	Kang (92)	1	1	1	1		1							
2016	Tinelli (93)													
2017	Clave (94)			1		1								
2017	Lim (95)	1												
2017	Satitniramai (96)	1		1				1	1					
2017	Singla (97)											1		
2017	Yaman Tunc (98)		1											
2018	Benson (99)													
2018	Blackwell (3)							1						
2018	Koroglu (100)		1											
2018	Li (101)													
2018	Petersen (102)													
2019	Inan (103)		1				1							
2019	Otkjaer (104)													
2019	Sirota (105)													
2019	Sondgeroth (106)													

†: due to parenchyma, fibroids or tumour. BMI: body mass index. LSCS: lower section caesarean section.

Appendix 7. Strategies to prevent urological injury

Year	First author	Anatomical knowledge (n= 12)	Strong uterine traction (n= 12)	Careful dissection generally (n= 9)	Prophylactically identify ureters (n= 8)	Distend bladder with fluid (n= 7)	Avoid diathermy near ureters (n= 6)	Dissect bladder from uterus † (n= 3)	Empty bladder with IDC (n= 3)	Prophylactic ureteric stents (n= 3)	Shield bladder with retractor (n= 2)
2000	Conde-Agudelo (12)										
2001	Cosson (13)		1	1				1	1		1
2001	Leanza (14)	1	1		1						
2001	Liapis (15)			1						1	
2001	Mathevet (16)	1		1							1
2001	Milad (17)										
2001	Seman (18)						1				
2002	Wattiez (19)		1								
2003	Boukerrou (20)										
2003	Khashoggi (21)										
2003	Shen (22)										
2004	Dorairajan (23)				1						
2004	Koh (24)	1			1						
2004	Parkar (25)										
2004	Rashid (26)										
2004	Steed (27)										
2005	Chang (28)										
2005	Phipps (29)										
2005	Tae Kim (30)										
2005	Vakili (31)			1							
2006	Akyol (32)										
2006	Bojahr (33)										
2006	Dauleh (34)										
2006	Ghezzi (35)										
2006	Kafy (36)										
2006	Mahdavi (37)										
2006	Roman (38)										
2006	Sharon (39)						1				
2007	Johnston (40)				1	1					
2007	Karaman (41)		1					1			
2007	Leonard (42)	1			1						
2007	Nawaz (43)	1	1								
2007	Ng (44)		1		1						
2007	O'Hanlan (45)		1		1	1					
2007	Soong (46)	1					1				
2007	Tian (47)										
2007	Xu (48)		1			1					
2008	Chen (49)					1	1				
2008	Kyung (50)					1			1		
2009	Ark (51)										
2009	Chopin (52)										
2009	Donnez (53)										
2009	Ibeanu (54)										
2009	Juillard (55)										
2009	Lafay Pillet (56)		1	1		1	1				
2009	Lee (57)		1								
2009	Rahman (58)							1			
2009	Yan (59)	1		1							
2010	Gungorduk (60)										
2010	Wang (61)										
2010	Wright (62)										
2011	Anpalagan (63)										
2011	Brummer (64)										
2011	Doganay (65)										
2011	Jung (66)										
2011	Kavallaris (67)										
2011	Song (68)										
2012	Alsharani (69)										
2012	Cho (70)										
2012	Grosse-Drieling (71)										
2012	Khan (72)										
2012	Kobayashi (73)				1						
2012	Lee (74)			1						1	
2012	Mueller (75)										
2012	Rao (76)	1							1		
2012	Sandberg (77)	1		1							
2012	Teerapong (78)										
2012	Tuuli (79)										
2013	Choi (80)										
2013	Makinen (81)										
2013	Sheth (82)										
2014	Dutta (83)	1	1								
2014	Han (84)	1								1	
2014	Nguyen (85)										
2014	Park (86)										
2014	Zia (87)			1							
2015	Garabedian (88)										
2015	Kaplanoglu (89)										
2015	Tan-Kim (90)										
2016	Dolanbay (91)										
2016	Kang (92)						1				
2016	Tinelli (93)										
2017	Clave (94)										
2017	Lim (95)										
2017	Satitniramai (96)	1									
2017	Singla (97)										
2017	Yaman Tunc (98)										
2018	Benson (99)										
2018	Blackwell (3)										
2018	Koroglu (100)										
2018	Li (101)										
2018	Petersen (102)										
2019	Inan (103)		1			1					
2019	Otkjaer (104)										
2019	Sirota (105)										
2019	Sondgeroth (106)										

†: rather than separate uterus and bladder with blunt traction. IDC: indwelling urethral catheter.

Appendix 8. Newcastle-Ottawa Quality Assessment Scale for included studies.

		1. Selection				2. Comparability	3. Outcome			Total
Year	First author	Representativeness of exposed cohort	Selection of non-exposed cohort	Ascertainment of exposure	Demonstration outcomes not present at start of study	Comparability of cohorts	Assessment of outcome	Was follow-up long enough for outcomes to occur?	Adequacy of follow up	
2000	Conde-Agudelo (12)	*	na	*	na	*	-	*	*	5
2001	Cosson (13)	*	na	-	na	-	-	-	-	1
2001	Leanza (14)	-	na	*	na	-	*	-	-	2
2001	Liapis (15)	-	na	-	na	-	-	-	-	0
2001	Mathevet (16)	-	na	-	na	-	-	-	-	0
2001	Milad (17)	*	na	*	na	*	*	-	-	4
2001	Seman (18)	-	na	*	na	-	*	-	-	2
2002	Wattiez (19)	*	na	*	na	**	*	-	-	5
2003	Boukerrou (20)	-	na	*	na	**	*	-	-	4
2003	Khashoggi (21)	*	na	*	na	**	*	-	-	5
2003	Shen (22)	-	na	*	na	*	*	-	-	3
2004	Dorairajan (23)	-	na	*	na	-	*	-	-	2
2004	Koh (24)	-	na	*	na	*	*	*	-	4
2004	Parkar (25)	*	na	*	na	*	*	-	-	4
2004	Rashid (26)	*	na	*	na	*	*	*	*	6
2004	Steed (27)	*	na	*	na	*	*	*	*	6
2005	Chang (28)	-	na	*	na	*	*	*	*	5
2005	Phipps (29)	-	na	*	na	**	*	-	-	4
2005	Tae Kim (30)	-	na	*	na	**	*	-	-	4
2005	Vakili (31)	*	na	*	na	**	*	*	*	7
2006	Akyol (32)	*	na	*	na	*	*	*	-	5
2006	Bojahr (33)	*	na	*	na	**	*	-	-	5
2006	Dauleh (34)	-	na	*	na	-	*	-	-	2
2006	Ghezzi (35)	*	na	*	na	**	*	*	-	6
2006	Kafy (36)	*	na	*	na	**	*	-	-	5
2006	Mahdavi (37)	-	na	*	na	**	*	-	-	4
2006	Roman (38)	-	na	*	na	*	*	-	-	3
2006	Sharon (39)	*	na	*	na	*	*	-	-	4
2007	Johnston (40)	-	na	*	na	**	*	-	-	4
2007	Karaman (41)	*	na	-	na	-	-	-	-	1
2007	Leonard (42)	*	na	*	na	**	*	-	-	5
2007	Nawaz (43)	-	na	*	na	-	*	-	-	2
2007	Ng (44)	-	na	-	na	**	-	-	-	2
2007	O'Hanlan (45)	-	na	*	na	**	*	-	-	4
2007	Soong (46)	-	na	*	na	-	*	*	*	4
2007	Tian (47)	*	na	*	na	-	*	-	-	3
2007	Xu (48)	-	na	*	na	-	*	*	-	3
2008	Chen (49)	-	na	-	na	*	-	*	*	3
2008	Kyung (50)	-	na	*	na	-	*	-	-	2
2009	Ark (51)	*	na	*	na	**	*	-	-	5
2009	Chopin (52)	*	na	*	na	**	*	-	-	5
2009	Donnez (53)	-	na	*	na	-	*	*	-	3
2009	Ibeanu (54)	*	na	*	na	**	*	-	-	5
2009	Juillard (55)	-	na	*	na	**	*	-	-	4
2009	Lafay Pillet (56)	*	na	*	na	**	*	-	-	5
2009	Lee (57)	*	na	*	na	*	*	*	-	5
2009	Rahman (58)	*	na	*	na	**	*	*	-	6
2009	Yan (59)	-	na	*	na	*	*	-	-	3
2010	Gungorduk (60)	*	na	*	na	**	*	*	-	6
2010	Wang (61)	*	na	*	na	-	*	-	-	3
2010	Wright (62)	*	na	*	na	*	*	-	-	4
2011	Anpalagan (63)	-	na	*	na	-	*	*	-	3
2011	Brummer (64)	*	na	*	na	-	*	-	-	3
2011	Doganay (65)	*	na	*	na	**	*	-	-	5
2011	Jung (66)	*	na	*	na	**	*	-	-	5
2011	Kavallaris (67)	-	na	*	na	**	*	-	-	4
2011	Song (68)	-	na	*	na	**	*	*	*	6
2012	Alsharani (69)	-	na	*	na	-	*	-	-	2
2012	Cho (70)	*	na	*	na	**	*	*	-	6
2012	Grosse-Drieling (71)	*	na	*	na	**	*	-	-	5
2012	Khan (72)	-	na	*	na	-	*	-	-	2
2012	Kobayashi (73)	*	na	*	na	**	*	-	-	5
2012	Lee (74)	-	na	*	na	-	*	*	-	3
2012	Mueller (75)	*	na	-	na	**	-	-	-	3
2012	Rao (76)	-	na	-	na	*	-	-	*	2
2012	Sandberg (77)	*	na	*	na	**	*	-	-	5
2012	Teerapong (78)	-	na	*	na	**	*	*	-	5
2012	Tuuli (79)	*	na	*	na	**	*	*	-	6
2013	Choi (80)	*	na	*	na	*	*	-	-	4
2013	Makinen (81)	-	na	*	na	**	*	-	-	4
2013	Sheth (82)	-	na	-	na	-	-	-	-	0
2014	Dutta (83)	-	na	*	na	-	*	-	-	2
2014	Han (84)	-	na	-	na	*	-	-	-	1
2014	Nguyen (85)	*	na	*	na	**	*	-	-	5
2014	Park (86)	*	na	*	na	**	*	*	*	7
2014	Zia (87)	*	na	*	na	*	*	-	-	4
2015	Garabedian (88)	-	na	*	na	**	*	*	*	6
2015	Kaplanoglu (89)	*	na	*	na	*	*	*	-	5
2015	Tan-Kim (90)	-	na	*	na	**	*	*	*	6
2016	Dolanbay (91)	-	na	*	na	*	*	-	-	3
2016	Kang (92)	*	na	-	na	*	-	*	-	3
2016	Tinelli (93)	*	na	*	na	**	*	*	*	7
2017	Clave (94)	-	na	*	na	**	*	*	-	5
2017	Lim (95)	-	na	*	na	*	*	-	-	3
2017	Satitniramai (96)	-	na	*	na	-	*	-	-	2
2017	Singla (97)	-	na	*	na	*	*	-	-	3
2017	Yaman Tunc (98)	*	na	*	na	*	*	-	-	4
2018	Benson (99)	*	na	*	na	-	*	-	-	3
2018	Blackwell (3)	*	na	*	na	*	*	*	*	6
2018	Koroglu (100)	*	na	*	na	**	*	-	-	5
2018	Li (101)	*	na	*	na	*	*	*	*	6
2018	Petersen (102)	-	na	*	na	**	*	*	-	5
2019	Inan (103)	*	na	*	na	**	*	-	-	5
2019	Otkjaer (104)	-	na	*	na	**	*	-	-	4
2019	Sirota (105)	*	na	*	na	*	*	-	-	4
2019	Sondgeroth (106)	*	na	*	na	*	*	-	-	4

Studies scoring 0-3, 4-5 and 6-7 points were identified as high, medium and low risk of bias, respectively. * indicates one point; ** indicates two points. Na: not applicable.
